# Discovery of the first dual GSK3β inhibitor/Nrf2 inducer. A new multitarget therapeutic strategy for Alzheimer’s disease

**DOI:** 10.1038/srep45701

**Published:** 2017-03-31

**Authors:** Isabel Gameiro, Patrycja Michalska, Giammarco Tenti, Ángel Cores, Izaskun Buendia, Ana I. Rojo, Nikolaos D. Georgakopoulos, Jesús M. Hernández-Guijo, María Teresa Ramos, Geoffrey Wells, Manuela G. López, Antonio Cuadrado, J. Carlos Menéndez, Rafael León

**Affiliations:** 1 Instituto Teófilo Hernando y Departamento de Farmacología y Terapéutica, Facultad de Medicina. Universidad Autónoma de Madrid, 28029 Madrid, Spain; 2 Instituto de Investigación Sanitaria, Servicio de Farmacología Clínica, Hospital Universitario de la Princesa, 28006 Madrid, Spain; 3 Departamento de Química Orgánica y Farmacéutica, Facultad de Farmacia, Universidad Complutense, 28040 Madrid, Spain; 4 Centro de Investigación Biomédica en Red sobre Enfermedades Neurodegenerativas (CIBERNED), Instituto de Investigación Sanitaria La Paz (IdiPaz), Instituto de Investigaciones Biomédicas Alberto Sols UAM-CSIC y Departamento de Bioquímica, Facultad de Medicina, Universidad Autónoma de Madrid, Madrid, Spain; 5 UCL School of Pharmacy, University College London, 29/39 Brunswick Square, London WC1N 1AX UK

## Abstract

The formation of neurofibrillary tangles (NFTs), oxidative stress and neuroinflammation have emerged as key targets for the treatment of Alzheimer’s disease (AD), the most prevalent neurodegenerative disorder. These pathological hallmarks are closely related to the over-activity of the enzyme GSK3β and the downregulation of the defense pathway Nrf2-EpRE observed in AD patients. Herein, we report the synthesis and pharmacological evaluation of a new family of multitarget 2,4-dihydropyrano[2,3-*c*]pyrazoles as dual GSK3β inhibitors and Nrf2 inducers. These compounds are able to inhibit GSK3β and induce the Nrf2 phase II antioxidant and anti-inflammatory pathway at micromolar concentrations, showing interesting structure-activity relationships. The association of both activities has resulted in a remarkable anti-inflammatory ability with an interesting neuroprotective profile on *in vitro* models of neuronal death induced by oxidative stress and energy depletion and AD. Furthermore, none of the compounds exhibited *in vitro* neurotoxicity or hepatotoxicity and hence they had improved safety profiles compared to the known electrophilic Nrf2 inducers. In conclusion, the combination of both activities in this family of multitarget compounds confers them a notable interest for the development of lead compounds for the treatment of AD.

Alzheimer’s disease (AD) is the most common neurodegenerative disease^1^, with a prevalence of more than 50 million cases worldwide in 2015, a number that is expected to reach 135 million in 2050. After more than a century of intensive research, the causes of AD are still largely unknown and consequently, the discovery of effective therapies remains a critical objective of modern medicine^2^. AD is characterized by the formation of intracellular neurofibrillary tangles (NFTs), composed of hyperphosphorylated tau protein, and extracellular amyloid-β plaques (Aβ), formed by amyloid-β peptide. Both hallmarks, along with extensive oxidative stress and chronic neuroinflammation, are considered major effectors of the complex AD neurodegenerative progression.

Several studies have demonstrated a direct correlation between the appearance of NFTs and the cognitive decline observed in AD^3^ suggesting that the tau component is a primary target in drug development programs in AD^4^. Glycogen synthase kinase-3β (GSK3β) is one of the most important kinases implicated in tau hyperphosphorylation, and it plays a pivotal role in the etiopathogenesis of AD^5^,^6^,^7^. GSK3β is over-expressed in the brain of AD patients, directly contributing to the formation of NFTs^8^. Furthermore, this kinase is also related to Aβ deposition^9^, oxidative stress and gliosis^5^. Recently, it has also been shown that GSK3β is a key mediator of apoptosis, thereby taking part in the mechanism involved in the neuronal loss in AD^10^. Thus, the search of GSK3β inhibitors has been extensively pursued and several compounds have reached clinical trials. However, the results have been disappointing either by lack of therapeutic action or side effects^11^ due to the wide range of physiological actions in which GSK3β is known to be involved^12^.

Oxidative stress is an important phenomenon clearly involved in the pathogenesis and progression of AD^13^,^14^. Oxidative stress is not only a consequence of the primary AD cascade of events, but also a cause of the initial onset of the disease^15^. It is present in the preliminary phase, known as mild cognitive impairment, when the Aβ plaques and the NFTs are not yet evident^16^. Furthermore, there are convergent neurotoxic effects of hyperphosphorylated tau, Aβ aggregates and oxidative stress whereas they induce and increase their reciprocal appearance in a positive feedback loop, intensifying neuronal damage and accelerating cognitive decline^17^,^18^. To counteract the harmful effects generated by oxidative stress, cells employ the nuclear factor erythroid 2-related factor 2/electrophile response element (Nrf2/EpRE) transcriptional pathway which promotes the synthesis of numerous antioxidant and anti-inflammatory enzymes^19^. Despite extensive evidence of high levels of oxidative stress in AD brains, Nrf2 is predominantly cytoplasmic in neurons, demonstrating the failure of this pathway^20^. Thus, the Nrf2-EpRE pathway has emerged as a promising pharmacological target for the treatment of AD^21^,^22^,^23^,^24^,^25^,^26^. Furthermore, there is evidence that the activities of GSK3β and Nrf2 are negatively correlated, thus increasing neuronal sensitivity to oxidative stress in AD^27^,^28^. Indeed, GSK3β is involved in the down-regulation of Nrf2 and control of its subcellular distribution^29^,^30^,^31^. In fact, several reports have demonstrated the interest in targeting GSK3β and Nrf2 as therapeutic strategies in AD^32^,^33^.

It is well established that both extensive oxidative stress and protein aggregates induce glial activation leading to chronic neuroinflammation^34^. Once activated, microglia produce pro-inflammatory cytokines, chemokines and free radical species, increasing oxidative stress, and thereby accelerating the neurodegenerative process. The chronic inflammatory status is also increased by the over-activity of GSK3β through several pathways^11^. It has also been demonstrated that neuroinflammation precedes and is sufficient to cause AD-like pathology^35^, implicating immune reactions early in the pathogenic process.

In this context, we were interested in finding a multitargeted drug combining two main activities: (1) GSK3β inhibition to diminish tau phosphorylation and to decrease cell death, improving neuronal survival and (2) Nrf2 induction properties, directed to reduce oxidative stress and the neuroinflammatory status. Furthermore, Nrf2 induction has proven to decrease the levels of phosphorylated tau protein by increasing the autophagy adaptor protein NDP52^36^. Therefore, the inclusion of both activities, GSK3β inhibition and Nrf2 induction, in a single molecule would reduce tau hyperphosphorylation by inhibiting the kinase and, at the same time, would help the cell to eliminate the aberrant hyperphosphorylated tau by facilitating its clearance. In this study, we present the synthesis, as well as the enzymatic and biological evaluation of the first class of compounds with dual activity as GSK3β inhibitors and Nrf2 inducers, providing a new therapeutic strategy against AD.

## Results and Discussion

**Figure 1:**
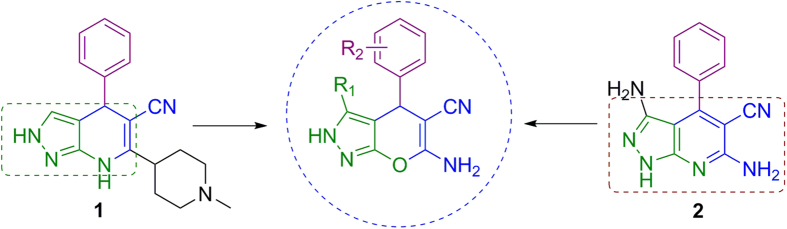
Selection of 2,4-dihydropyrano[2,3-*c*]pyrazole compounds based on structural features observed in known GSK3β inhibitors (1 and 2).

Based on the chemical structure of the GSK3β inhibitors **1**^37^ and **2**^38^ (Fig. 1), we thought that compounds bearing the 1,4-dihydropyridine core or the isostere 4*H*-pyran could be suitable as GSK3β inhibitors and we searched for their potential Nrf2 induction. We selected several compounds from our “in house” chemical library^39^,^40^,^41^,^42^ and screened them as potential GSK3β inhibitors and Nrf2 inducers. Successfully, we found that compounds bearing the 6-amino-2,4-dihydropyrano[2,3-*c*]pyrazole-5-carbonitrile (Fig. 1) were able to inhibit GSK3β and to induce Nrf2 at micromolar concentrations. These preliminary results prompted us to synthetize a small chemical library using this structural core in order to establish potential structure-activity relationships for future hit-to-lead optimization.

### Chemistry

**Figure 2:**
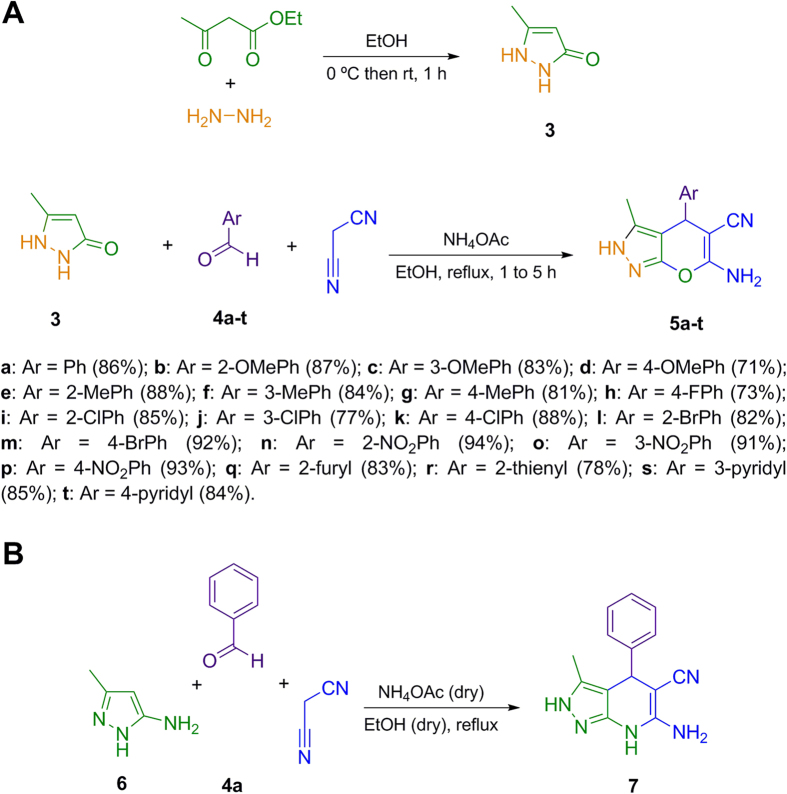
(**A**) Scope of the three-component synthesis of pyrano[2,3-*c*]pyrazoles **5a–t**. (**B**) Three-component synthesis of 6-amino-3-methyl-4-phenyl-4,7-dihydro-2*H*-pyrazolo[3,4-*b*]pyridine-5-carbonitrile 7.

The synthesis of a 2,4-dihydropyrano[2,3-*c*]pyrazole library was achieved *via* a variation of a known three-component reaction^43^,^44^ between 5-methyl-1*H*-pyrazol-3(2*H*)-one **3** (previously synthesized by condensation of hydrazine and ethyl acetoacetate), aromatic aldehydes **4a–t** and malononitrile, in refluxing ethanol, employing ammonium acetate as promoter of the reaction (Fig. 2).

This method allowed the preparation in good to excellent yields of the compounds **5**, bearing H (**5a**), electron-donating (**5b–g**) or electron-withdrawing (**5h–p**) groups at different positions on the aldehyde aromatic ring. To introduce further structural diversity in the 2,4-dihydropyrano[2,3-*c*]pyrazole derivatives, we also synthesized compounds bearing heterocyclic moieties at the C-4 position (**5q–t**).

We synthesized also the corresponding dihydro-2*H*-pyrazole[3,4-*b*]pyridine analogue **7** for comparison purposes. The dihydropyridine derivative **7** was obtained using the same three-component protocol from 3-methyl-1*H*-pyrazol-5-amine **6**, benzaldehyde **4a** and malononitrile in refluxing dry ethanol, using dry ammonium acetate as a promoter of the reaction (Fig. 2B).

### Pharmacology: Dual target study: GSK3β inhibition and Nrf2 induction ability of compounds 5a–t and 7

Compounds **5a–t** were tested as potential GSK3β inhibitors using a luminescence Kinase-Glo assay. All compounds were assayed at four concentrations (0.1, 1, 10 and 30 μM) and IC_50_ values and their respective apparent equilibrium dissociation constant (*K*_i_) values are summarized in Table 1. The known GSK3β inhibitor SB216763 was included as a positive control and compound **2** for comparative purposes. Inhibitory potencies ranged from 3.77 μM of compound **5m** (*p*-Br) to 21.4 μM of compound **5f** (*m*-Me). Regarding the substituents on the aromatic ring at the C-4 position, compounds bearing electron-withdrawing substituents were generally more potent inhibitors than those with an electron-donating substituent. Taking into account the position of the substituent, the inhibitory potency increased in the following way: *ortho* < *meta* < *para*. For example, compounds **5b**, **5c** and **5d** bearing an *o*-OMe, *m*-OMe and *p*-OMe substituents respectively, increased their inhibitory potency from 16.4 μM (*ortho*-position) to 7.20 μM (*meta*-position) and to 6.60 μM (*para*-position). Interestingly, this behaviour was reversed in the case of the nitro compounds, where the *ortho*-substituted derivative **5n** was the most potent (IC_50_ = 3.89 μM), followed by the *meta* (**5o**, IC_50_ = 7.20 μM) and the *para*-substituted derivatives (**5p**, IC_50_ = 16.3 μM). This inversion of the inhibitory tendency might be related to a different interaction pattern with the enzyme.

Considering the derivatives with a heterocyclic substituent, some correlations can be drawn. The replacement of the oxygen atom in derivative **5q** (2-furyl) by sulfur **5r** (2-thienyl) reduced the inhibitory potency more than two fold, from 14.8 μM to over 30 μM and the change of the nitrogen position in the pyridine ring from 3 (**5s**) to 4 (**5t**) improved the inhibitory capability more than 3.6 times.

Finally, the dihydropyridine derivative **7** showed an IC_50_ value of 18.6 μM, demonstrating an improved inhibitory activity compared to its phenyl analogue **5a**. The enhanced inhibitory activity of **7** is undoubtedly related to the inclusion of the NH moiety into the six-member ring.

Thereafter, we tested the ability of the compounds to induce the Nrf2 transcription factor. We used an Nrf2-dependent luciferase reporter assay in the AREc32 cell line^45^. Cells were cultured for 24 h and then treated with increasing concentrations of compounds **5a–t** (0.3, 3, 10 and 30 μM), including the known inducer sulforaphane, containing an isothiocyanate motive as positive control. As shown in Table 1, almost all compounds were able to significantly increase luciferase activity, with CD (concentration required to double the basal luciferase reporter activity) values ranging from 2.5 μM (**5t**, 4-pyr) to over 30 μM (ortho-substituted derivatives and **7**).

Regarding SAR analysis, in general, compounds bearing electron-withdrawing groups were more potent inducers than those with an electron-donating group. Comparing the different positions on the aromatic ring at C4, Nrf2 induction properties improved from *ortho-* to *para-* and *meta-,* with *meta*-derivatives being the most potent inducers. In all cases, *ortho* derivatives were poor Nrf2 inducers with CD values over 30 μM. Interestingly, in this experiment the nitro derivatives exerted a different behavior, demonstrated by the CD value of the *o*-NO_2_ derivative (**5n**, CD = 11.3 μM), being slightly more potent than the *m*-NO_2_ derivative (**5o**, CD = 18.9 μM).

The *meta* derivatives were, except for the nitro derivative, better inducers; being compound **5j** being the most potent of the phenyl-substituted derivatives (CD = 5.38 μM). The electronic effect of the substituent at the *para*- position was very important, as compounds bearing electron-donating substituents, namely **5d** (*p*-OMe) and **5g** (*p*-Me), were poor Nrf2 inducers (CD = 25.3 and 25.4 μM, respectively). On the other hand, electron-withdrawing derivatives, **5h** (*p*-F), **5k** (*p*-Cl), **5m** (*p*-Br), **5p** (*p*-NO_2_), were significantly more potent, showing CD values between 1.7 and 3 fold more potent than electron-donating *para-* substituted derivatives. Finally, looking at the heterocycle-substituted derivatives, compound **5t** bearing a 4-pyridyl substituent was the most potent inducer with a CD value of 2.5 μM.

Interestingly, the fused dihydropyridine **7** was completely unable to induce Nrf2, even at higher concentrations (60 and 100 μM) (see Supporting Information), although it was a better GSK3β inhibitor than its dihydropyrano[2,3-*c*]pyrazole analogue compound **5a**. This remarkable result indicates that the Nrf2 induction capability of the dihydropyrano[2,3-*c*]pyrazole derivatives **5a–t** is an intrinsic property of the core structure.

### Proposed binding pose of 5m into the ATP binding site of GSK3β

**Figure 3:**
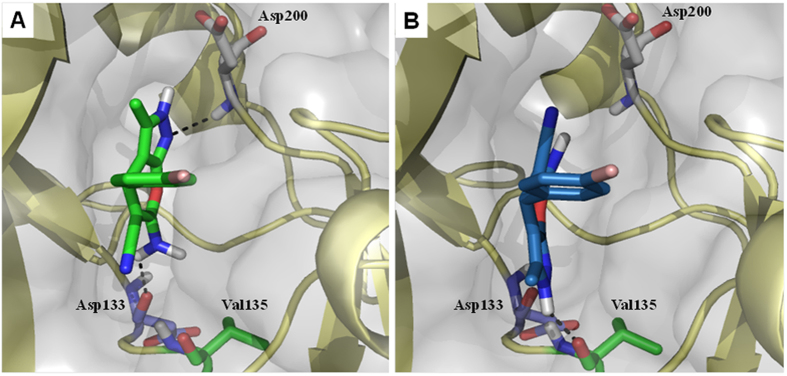
Proposed binding mode of 5m into ATP-binding site of GSK3β. Docking studies of the *p*-bromo derivative **5m** (*R* enantiomer, green sticks, panel A; *S* enantiomer, dark blue sticks, panel B) at the ATP binding site of GSK3β.

In order to obtain a deeper insight into the molecular interactions of compounds **5a–t** with GSK3β at the atomic level, we performed docking calculations. To this end, we selected the most potent GSK3β inhibitor found in this family to study its potential binding mode. Docking studies were performed with the AutoDock Vina program using the ATP binding site of GSK3β (PDB code 1PYX). We studied both enantiomers of compound **5m**, which showed similar poses and modes of interaction with a 180° flip position of the dihydropyrano[2,3-*c*]pyrazole core from *R* to *S* enantiomers. Thus, the best docking solutions for the dihydropyrano[2,3-c]pyrazole core of the *R* enantiomer shows a hydrogen bond of the N atom of the pyrazole ring to the backbone peptide bond of Asp200 and another H-bond contact between the carbonyl group of Asp133 and the amino moiety of the inhibitor (Fig. 3A). Conversely, docking solutions of *S* enantiomer of compound **5m**, which is flipped 180°, predict that the NH group of the pyrazole ring forms a hydrogen bonding interaction to the backbone carbonyl of Val135 and that the N atom of the same ring H-binds to the backbone NH of Val135 (Fig. 3B).

### Nrf2 induction properties are independent of the GSK3β inhibitory capability

**Figure 4:**
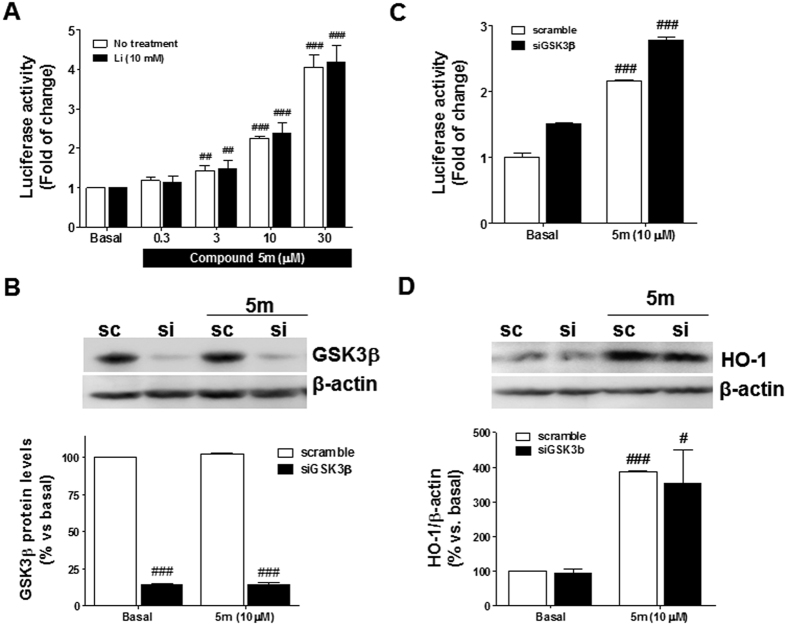
Nrf2 induction properties are independent of the GSK3β inhibitory activity. (**A**) Luciferase activity induced by compound **5m** alone and in presence of lithium compared to basal conditions. (**B**) GSK3β silencing efficiency achieved by treating AREc32 cells with 25 nM of siRNA and 2 μL of DharmaFECT1 during 48 h. (**C**) Luciferase activity induced by compound **5m** (10 μM) in AREc32 cells after 24 h. Cells were previously treated with scramble (white) or siGSK3β (black) silencing RNAs during 48 h. (**D**) HO-1 expression induced by compound **5m** (10 μM) measured at the same experimental conditions used previously. Values are expressed as mean ± SEM of three independent experiments in duplicate. ^###^p < 0.001; ^##^p < 0.01; ^#^p < 0.05 compared to the correspondent basal conditions.

Under stress conditions, GSK3β is over-activated and down-regulates Nrf2^27^, thus GSK3β inhibition can lead to Nrf2 induction as previously demonstrated^27^,^46^,^47^. However, in our experimental conditions GSK3β inhibition did not induce luciferase expression in the AREc32 cell line as demonstrated by the inability of the GSK3β inhibitor SB216763 to induce Nrf2 transcriptional activity (see Supporting Information). This result might be explained by the existence of several autocrine pathways that limit the effect of GSK3β over Nrf2 in this particular cell line^48^. To further evaluate the potential relationship between GSK3β inhibition and the Nrf2 induction properties we used two different approaches. Firstly, we used a pharmacological approach to inhibit the enzyme by using lithium, a well-known GSK3β inhibitor able to inhibit GSK3β activity with a Ki of 2 μM^49^. Incubation with Li (10 mM) inhibits almost completely GSK3β activity allowing us to evaluate the Nrf2 induction capability of our derivatives after pharmacological ablation of GSK3β^49^. We selected compound **5m** as the most potent GSK3β inhibitor to be assessed under these conditions. Cells were treated with compound **5m** at increasing concentrations alone or in presence of Li (10 mM) during 24 h and thereafter, luciferase activity was evaluated. As shown in Fig. 4A, compound **5m** was able to increase luciferase activity from 3 μM to 30 μM when it was incubated alone or in presence of Li (10 mM) to the same extent and without statistically significant differences. Secondly, to corroborate the independence of the Nrf2 induction properties of our derivatives from their GSK3β inhibitory properties, we used GSK3β knock down experimental conditions using siRNA. Knockdown of GSK3β by 85.7% according to western blot analysis (Fig. 4B) did not affect the Nrf2 induction potency of compound **5m**. After knockdown of GSK3β, AREc32 cells were incubated with compound **5m** (10 μM) during 24 h (Fig. 4B). Subsequently, luciferase activity and HO-1 expression were analyzed. As shown in Fig. 4C, knockdown of GSK3β moderately increased the activity of luciferase. Compound **5m** (10 μM) increased luciferase activity 2.2-fold compared to basal conditions in cells previously treated with scramble control siRNA. Similarly, **5m** was also able to increase luciferase activity 2.8-fold compared to basal conditions (Fig. 4C) when GSK3β was silenced. Similar results were obtained when HO-1 expression was analyzed (Fig. 4D), compound **5m** increased HO-1 expression 3.9 times after scramble treatment and 3.5 times after knockdown of GSK3β showing no statistical differences between both conditions. These results demonstrate that the Nrf2 induction mechanism of compound **5m** is independent of its GSK3β inhibitory properties. Thus, both mechanisms of action are complementary and independent.

Recently, inhibitors of the Nrf2-Keap1 protein-protein interaction have been reported as potent non-electrophilic Nrf2 inducers^50^, and thus, we tested such inhibition as a potential mechanism of action. Unfortunately, fluorescence polarization and differential scanning fluorimetry assays indicated that compounds **5a–t** are not able to inhibit the Nrf2-Keap1 interaction (see the Supporting Information). Therefore, further studies will be undertaken in order to elucidate the exact mechanism of action involved in the Nrf2 induction.

### Compounds 5a–t exert neuroprotection against Tau hyperphosphorylation and rotenone/oligomycin induced cytotoxicity

**Figure 5:**
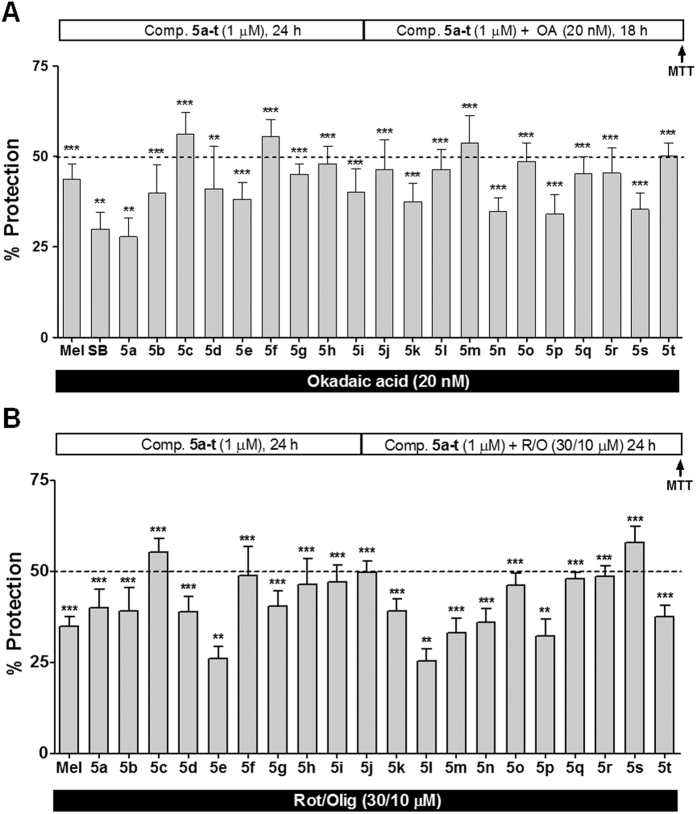
Compounds 5a–t exert neuroprotection in *in vitro* models of AD. Neuroprotective effect of compounds **5a–t** at 1 μM, melatonin (1 μM) and SB216763 (1 μM) against toxicity elicited by tau hyperphosphorylation (**A**, OA, 20 nM) or oxidative stress and energy depletion (**B**, R/O, 30/10 μM). SH-SY5Y cells were pre-incubated for 24 h with compounds **5a–t**, melatonin or SB216763 at 1 μM. Then, treatments were removed and cells were co-incubated with each treatment and okadaic acid for 18 h or R/O for 24 h. Cell viability was measured by the MTT reduction assay. Values are expressed as mean ± SEM of five independent experiments in triplicate. ***p < 0.001; **p < 0.01 respect to toxic stimuli treated cells, considered as 0% of protection.

As described above, tau hyperphosphorylation and the presence of neurofibrillary tangles is one of the central hallmarks in AD, and the main kinase related to tau hyperphosphorylation in AD is GSK3β. We selected a specific *in vitro* model of AD to test the potential neuroprotective activity of these derivatives against tau hyperphosphorylation. Okadaic acid (OA) exerts its toxicity by selectively inhibiting protein phosphatases 1 and 2A^51^, inducing tau-hyperphosphorylation and aggregation^52^. Furthermore, it also increases oxidative stress leading to cell death^53^,^54^. Results are expressed as percentage of protection, meaning the increase in cells survival compared to the toxicity induced by OA. In all experiments, the neuroprotective agent melatonin, and the GSK3β inhibitor SB216763 were included for comparative purposes (Fig. 5A).

In general, all tested compounds showed neuroprotective activities at 1 μM concentration, with protection percentages ranging from 27.7% for compound **5a** (Ph) to 56.2% for compound **5c** (*m*-OMe). Among them, compounds **5c** (*m*-OMe), **5f** (*m*-Me), **5m** (*p*-Br) and **5t** (4-pyridyl) showed neuroprotective activities over 50%. Compared to reference compounds, most derivatives were able to protect neuroblastoma cells better than melatonin and SB216763, although they are less potent GSK3β inhibitors than the latter, proving that their neuroprotective effect also depends on Nrf2 induction. This observation is in agreement with the fact that OA induced toxicity depends on tau hyperphosphorylation and oxidative stress. From SAR studies, the inclusion of substituents at the aromatic ring improved their protective activity in all cases, using as a reference phenyl derivative **5a**, which showed the poorest neuroprotective effect as well as poor GSK3β inhibition (>30 μM) and poor Nrf2 induction properties (CD = 20.5 μM). The general behavior was the enhancement of the protective capabilities when the GSK3β inhibitory potency and the Nrf2 induction potency increased. This is particularly evident in the case of compounds **5c** (*m*-OMe), **5m** (*p*-Br) and **5t** (4-pyridyl), which showed protection percentages over 50%, are among the most potent GSK3β inhibitors (IC_50_ lower than 10 μM) and Nrf2 inducers (CD lower than 10 μM for **5m** and **5t**). Similarly, derivative **5c** showed a similar potency to compound **5d** as a GSK3β inhibitor, but **5c** was 2.2 times better as an Nrf2 inducer, and showed the highest neuroprotective activity (56.2%) against OA.

However, this trend was different in the case of the nitro derivatives, as also observed for the inhibition of GSK3β and Nrf2 induction. In this case, compound **5n** (*o*-NO_2_) had the highest inhibitory potency (IC_50_ = 3.9 μM) among nitro derivatives and good Nrf2 induction properties, showed a lower neuroprotective effect (34.7%) than its *m*-NO_2_ substituted analogue **5o** (48% protection), that was a two-fold poorer GSK3β inhibitor and also a less potent Nrf2 inducer.

As stated in the Introduction, oxidative stress is considered one of the main causes of neuronal death in AD. Therefore, we were interested in evaluating the potential antioxidant activity of compounds **5a–t** in a model of oxidative stress and energy depletion, which in turn increase cell death. For this purpose, we selected the combination of rotenone (30 μM) and oligomycin A (10 μM) (R/O) to induce neurotoxicity as a model system^23^,^40^ to evaluate the potential neuroprotective activity of the compounds. Melatonin, a well-known antioxidant natural compound, was included as a positive control.

As shown in Fig. 5B, compounds **5a–t** exhibited interesting neuroprotective properties against the cytotoxicity induced by the combination of R/O, with neuroprotection values ranging from 24.1% of compound **5l** (*o*-Br) to 57.9% of compound **5s** (3-pyridyl). Importantly, almost all derivatives showed a greater neuroprotective effect than melatonin. In general, the antioxidant neuroprotective effect was proportional to the Nrf2 induction capability that, in turn, depends on the type of substituent and its position on the aromatic ring.

As described for Nrf2 induction, the antioxidant neuroprotective activity increased from *ortho-* to *para-* and *meta-* derivatives, following the same trend as the Nrf2 induction properties. For example, compounds **5b** (*o*-OMe) and **5d** (*p*-OMe), were poor Nrf2 inducers and both showed similar neuroprotective effects (39.1% and 38.8%, respectively). Instead, compound **5c**, bearing a *meta-*methoxy substitution showed the best Nrf2-inducer capability among methoxy derivatives, and showed an improved neuroprotective effect (55.3%), being the second best neuroprotectant agent of the family. This tendency was reproduced for methyl, chloro and nitro substituted derivatives. Finally, the 3-pyridyl analogue, the second most potent Nrf2 inducer, was the best neuroprotectant agent against the R/O combination, being able to reduce 57.9% of cell death.

### Compounds 5a–t reduced nitrite and ROS production in primary glial cultures stimulated with lipopolysaccharide

The overexpression and/or increased activity of GSK3β have been widely associated with increased microglial activation in AD, leading to cell death^55^. On the other hand, the deregulation of the Nrf2-EpRE pathway in AD^20^ leads to a situation of exacerbated oxidative stress that also induces glial activation. Finally, activated glial cells liberate different pro-inflammatory cytokines (TNFα and IL1β, among others) and induce the activation of NADPH oxidase (NOX) and the over-expression of the inducible nitric oxide synthase (iNOS), increasing the levels of oxidative stress and enhancing oxidative damage. The cross-link between GSK3β, Nrf2 and the production of free radicals generates a positive auto-regulated loop, leading to a situation of chronic inflammation that has been widely described as a pathological hallmark of AD.

Therefore, we envisaged the study of compounds **5a–t** as potential anti-inflammatory agents. We used primary rat glial cells treated with lipopolysaccharide (LPS) to induce the production of nitrites and ROS as a model of glial activation. Nitrite production is directly proportional to the expression of the iNOS, that is over-expressed when LPS activates glial cells. ROS production is proportional to NOX activation after stimulation with LPS. Results are summarized in Table 2 as IC_50_s of nitrite production reduction and ROS production reduction. We have included sulforaphane as control in all experiments (see the Supporting Information).

In terms of substituent position, the capacity to reduce nitrite release improved from *ortho* to *para* and *meta,* following a similar tendency to Nrf2 induction (Table 1). In general, the variation of the anti-inflammatory activity was dependent on the position and the steric hindrance of the substituent, an observation that confirms the link between the anti-inflammatory effect with enzyme inhibition potency and the Nrf2 induction properties. Regarding the substituents, in the case of the methoxy derivatives the *ortho*-derivative **5b** showed a poor anti-inflammatory effect, correlating with moderate enzyme inhibition (IC_50_ = 16.4 μM) and a poor Nrf2 induction (CD > 30 μM). When the methoxy substituent was moved to *para*-position (**5d**), anti-inflammatory potency was slightly increased, an improvement that correlates with an inhibitory activity 2.5-fold more potent (IC_50_ = 6.60 μM) and a slightly improved Nrf2 induction effect (CD = 25.3 μM). This observation was more evident in the case of the *m*-OMe derivative, which was 2.3 times better as a GSK3β inhibitor (IC_50_ = 7.20 μM) compared to the *ortho*-derivative, and also a better Nrf2 inducer (CD = 11.3 μM). This behavior was repeated for electron-withdrawing substituents, such as chlorine and bromine substituents.

The nitro derivatives **5n** (*o*-NO_2_), **5o** (*m*-NO_2_) and **5p** (*p*-NO_2_) deserve a special mention since they followed an opposite tendency, as described above for the GSK3β inhibition and Nrf2 induction properties. For this substituent, the poorest anti-inflammatory activity was found for compound **5o**, bearing an *m*-nitro substituent. This result correlates with a moderate inhibitory activity (IC_50_ = 7.20 μM) and medium Nrf2 induction properties. Finally, while most *ortho-*substituted derivatives demonstrated to be poor anti-inflammatory agents, the *o*-nitro substituted derivative showed good anti-inflammatory effect. Compound **5n** (*o*-NO_2_) showed the best GSK3β inhibitory activity (IC_50_ = 3.89 μM) and a potent Nrf2 induction capability, being the most active of the three -NO_2_ analogues in both biological activities. This observation further confirms the dependence of the anti-inflammatory effect on both targets.

Besides the increase in nitrite production, it is well known that LPS stimulation of microglia also induces the activation of NOX, which induces superoxide free radical production^56^. In fact, this mechanism is synergistic with the expression of iNOS and the production of nitrites. In this respect, there is a body of evidence showing that NOX activation is a major contributor to ROS oxidative stress and neuronal death in AD^57^,^58^. Thus, we deemed it of interest to demonstrate whether compounds **5a–t** were also able to reduce ROS production upon stimulation with LPS. As shown in Table 2, most of the compounds successfully reduced ROS production measured by using the ROS sensitive dye H_2_DFCDA (see Supporting Information). In this case the reduction of ROS production afforded by the compounds showed a similar trend to that observed in the nitrite production assay; however, the IC_50_s were higher for this assay when compared to the nitrite values. In general, potency to reduce ROS production increased from *ortho* to *para* and *meta* as observed above. Similarly, nitro derivatives **5n** (*o*-NO_2_), **5o** (*m*-NO_2_) and **5p** (*p*-NO_2_) showed an inversion of the general tendency being compound **5o** (*m*-NO_2_, IC_50_ = 30.8 μM) less potent than the corresponding *ortho*-derivative **5n** (IC_50_ = 19.9 μM). These results further confirm the relationships observed in the case of nitrite reduction on the dependency of both mechanism of action exerted by compounds **5a–t**.

### Compound 5c reduced TNFα production, NOX activity, NOX2 and iNOS expression induced by LPS and induced HO-1 expression in glial cultures

**Figure 6:**
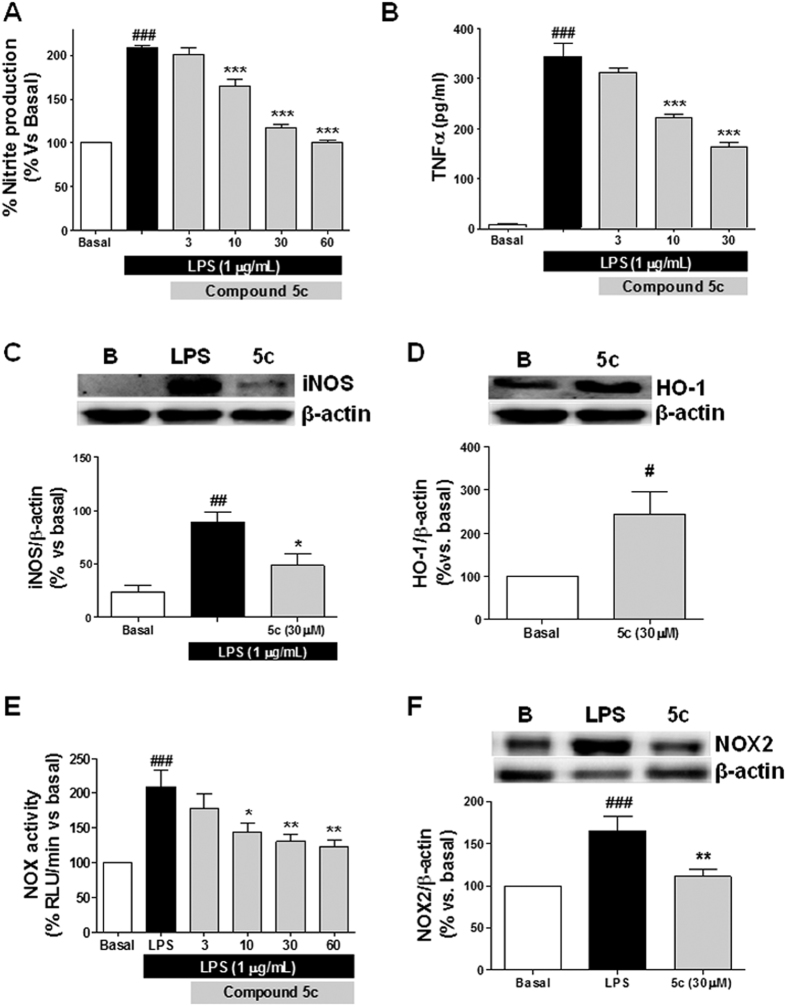
Nitrites (**A**), TNFα (**B**), iNOS (**C**) and NOX activity (**E**) blockade (**D**) and NOX2 production (**F**) by 5c in primary glial cultures stimulated with LPS (1 μg/mL), HO-1 expression induced by this compound after 24 h. Primary glial cells cultures were treated with compound 5c at increasing concentrations (3, 10, 30 and 60 μM) for 24 h, thereafter, treatments were removed and glial cells were co-incubated with compound 5c at increasing concentrations and LPS (1 μg/mL) for 18 h more. To measure NOX2 and HO-1 expression glial cultures were treated with compound 5c (30 μM) for 24 h. NOX activity was measured after 4 h co-treatment with LPS and compound 5c. Data are means ± SEM of four different experiments in triplicate. ^###^p < 0.001 compared to basal levels; ***p < 0.001; **p < 0.01; *p < 0.05 compared to LPS condition.

To further demonstrate the anti-inflammatory capacity of these compounds, we measured the most important mechanism of ROS production related to neuroinflammation (NOX activity, NOX2 and iNOS expression) and the TNFα release induced by LPS as markers of glial activation. TNFα is considered one of the most important pro-inflammatory cytokines. For this study, we selected compound **5c** (Ar = *m*-OMeC_6_H_4_) that showed good properties as a GSK3β inhibitor and Nrf2 inducer, and the best neuroprotective profile in both neuroprotection models. Furthermore, **5c** significantly reduced nitrite production in a concentration dependent manner (Fig. 6A). Interestingly, as shown in Fig. 6, compound **5c** was able to block the production of TNFα (Fig. 6B) in a concentration-dependent manner and to reduce the expression of the iNOS enzyme at 30 μM (Fig. 6C) almost completely. The production of TNFα induced by LPS was reduced by 33% at 10 μM and by 50% at 30 μM. Interestingly, we also demonstrated that compound **5c** was able to increase the expression of HO-1 2.3 times in glial cultures (Fig. 6D). As described above, LPS stimulation induces the activation of NOX enzyme to produce high amounts of superoxide; compound **5c** was able to significantly reduce the activity of NOX (p < 0.01) in a concentration dependent manner from 10 to 60 μM (Fig. 6E) measured after 4 h stimulation with LPS (1 μg/mL). To further confirm the anti-inflammatory potential of compound **5c** we measured the expression of thre NOX2 enzyme, since this isoform has been widely described as being specific tomicroglial cells^59^,^60^. As depicted in Fig. 6F, LPS (1 μg/mL) stimulation of primary glial cultures during 24 h increased the expression of NOX2 by 65.3% (p < 0.01) compared to basal conditions. Finally, compound **5c** (30 μM) abolished almost completely (11.1% increase; p < 0.001) the over-expression of NOX2 induced by LPS (p < 0.01).

### Toxicological evaluation

In general, Nrf2 inducers are electrophilic compounds, which tend to be hepatotoxic, limiting their therapeutic use. Furthermore, hepatotoxicity is one of the major reasons for drug withdrawals in clinic^61^,^62^,^63^,^64^,^65^. Thus, it is relevant to study the potential neurotoxicity and hepatotoxicity of the novel dual GSK3β inhibitors-Nrf2 inducers **5a–t**. As shown in Table 3 of the Supporting Information, none of the described compounds revealed a significant toxicity in any of the cell lines used, namely the SH-SY5Y neuroblastoma cell line and the HepG2 hepatoblastoma cell line. In both cell lines, all compounds demonstrated low toxicity, with LD_50_ values over 100 μM, showing a good safety profile. Compared to the potent Nrf2 inducer sulforaphane (SH-SY5Y, LD_50_ = 13.3 μM; HepG2, LD_50_ = 9.9 μM), all these derivatives were, at least, 10 times less toxic.

## Conclusions

In summary, we report here the first examples of compounds with dual activity as GSK3β inhibitors - Nrf2 inducers as a multitarget approach for the treatment of AD. This combination of activities represents an interesting and novel approach against AD. Our data show that the Nrf2 induction capability of these compounds is an intrinsic pharmacological property of the 2,4-dihydropyrano[2,3-*c*]pyrazole core and is independent of GSK3β inhibition, as demonstrated by: (1) a representative example of its dihydropyridine-fused isosteric framework (compound **7)** was completely unable to induce Nrf2; (2) pharmacological ablation of GSK3β activity did not abolish Nrf2 induction capacity of compound **5m**; and (3) knockdown of GSK3β with siRNA did not affected this capacity either. Further studies to demonstrate the mechanism of action by which these compounds are able to induce the activation of the phase II antioxidant response are under development in our laboratory and will be reported in due course. Furthermore, the combination of activities achieved led to interesting structure-activity relationships linking both properties with their neuroprotective and anti-inflammatory capabilities. These relationships will enable us to undertake a future hit-to-lead optimization process to obtain a lead molecule with an improved pharmacological profile.

In conclusion, the combination of GSK3β inhibition and Nrf2 induction in one molecule, described here for the first time, is aimed at decreasing tau hyperphosphorylation, to reduce oxidative stress and to diminish chronic neuroinflammation, that are major pathological hallmarks of AD.

## Methods

### Chemistry

All reagents (Aldrich, Fluka, SDS, Probus) and solvents (SDS) were of commercial quality and were used as received. Reactions were monitored by thin layer chromatography using commercially available aluminum-backed plates coated with silica gel Scharlau Cf 530 with fluorescent indicator and visualized under ultraviolet light lamp Camag UV-II (at 254 and 366 nm). Flash column chromatography was carried out using silica gel SDS 60 ACC or Scharlau Ge 048 and the eluent indicated in each case. Automatic flash chromatography was performed in a Teledyne ISCO COMBI *Flash* Rf instrument using Redi*Sep* Rf silica columns (4 g and 12 g) or self packed silica cartridges. Melting points were determined using a Stuart Scientific apparatus, SMP3 Model, and are uncorrected. All compounds were characterized using spectroscopic data and HRMS. The purity of new compounds was assessed by CHNS elemental analysis, and all values were verified to be within ±0.4% of the theoretical values. Infrared spectra were recorded on an Agilent Cary630 FTIR spectrometer with a diamond ATR accessory for solid and liquid samples, requiring no sample preparation. NMR spectra were obtained on a Bruker Avance 250 spectrometer operating at 250 MHz for ^1^H and 63 MHz for ^13^C (CAI de Resonancia Magnética Nuclear, Universidad Complutense). Gas chromatography mass spectra (GC-MS) were taken with different ionization methods, as Electronic Impact (EI) or Electrospray Ionization (ESI) in both the positive and negative ion mode, and were carried out by SIDI from Universidad Autónoma de Madrid (Madrid, Spain). Quantitative elemental analysis by combustion of carbon, hydrogen, nitrogen and sulfur were carried out in Servicio de Microanálisis Elemental from Universidad Complutense (Madrid, Spain), using a Leco CHNS 932 Elemental Analyzer.

### General procedure for the synthesis of 2,4-dihydropyrano[2,3-c]pyrazole derivatives 5a–t

A solution of pyrazolone derivative **3** (1 equiv, 1 mmol), malononitrile (1 equiv, 1 mmol), the corresponding arylaldehyde **4a–t** (1 equiv, 1 mmol) and ammonium acetate (1 equiv, 1 mmol) in ethanol (2 mL) was heated under reflux for 5 hours. After this time the solvent was evaporated under reduced pressure and the crude residues were crystallized from EtOH or purified by silica gel column chromatography using dichloromethane:methanol mixtures as eluent to give pure compounds (**5a–t**). For characterization data for all final compounds; see the Supporting Information.

## Pharmacology

### GSK3β inhibition

GSK3β inhibitory activity was evaluated using the method developed by Baki^66^, with modifications. Compounds were dissolved in assay buffer, containing 40 mM Tris (pH 7.5), 20 mM MgCl_2_, 0.1 mg/ml BSA (bovine serum albumin) and 50 μM dithiothreitol (DTT) at the desired concentrations. Firstly, 10 μl of enzyme (10 ng) and 10 μl of each compound were mixed for 30 min. Then, 20 μl of a mixture of ATP (1 μM) and GSK3β peptidic substrate were added to each well. The mixture was incubated for 60 min at 30 °C. Thereafter, the remaining ATP concentration was measured with the Kinase-Glo system following instructions from the supplier. Luminescence was measured in an Orion II microplate luminometer (Berthold, Germany) as relative light units (RLU). GSK3β activity is proportional to the difference between total ATP and remaining ATP after the enzymatic reaction, activity was considered to be 100% in the absence of an inhibitor. IC_50_ values were calculated by non-linear regression analysis of individual concentration-response curves using GraphPad Prism 5.0 software (San Diego, CA, USA).

### Luciferase activity: Nrf2 induction

AREc32 cells were plated in 96-well white plates (2 × 10^4^ cells/well). After 24 h, cells were incubated with increasing concentrations of each compound in duplicate for 24 h. AREc32 cells express constitutively the plasmid pGL-8xARE that implements 8 copies of the EpRE sequences followed by luciferase reporter gen. Therefore, Nrf2 induction is related to the activation of EpRE sequences, expressing luciferase at the same extent as EpRE sequences are activated. The Luciferase Assay System (Promega E1500), was used according to provider protocol and luminescence was quantified in an Orion II microplate luminometer (Berthold, Germany). Fold induction of luciferase activity was normalized to basal conditions. Data are expressed as CD values, expressing the concentration required to double the luciferase activity. CD values are calculated from dose-response curves generated from fold induction of control conditions *vs.* inducer concentration and fitted by non-linear regression and data interpolated to 2-fold induction concentration.

### Docking calculations

The GSK3β crystallographic structure PDB ID 1PYX containing phosphoaminophosphonic acid-adenylate ester (AMP-PNP), was used to perform the molecular docking. To test our system, we first performed the docking of ANP at the ATP-binding site using AutoDock Vina (version 1.1.2). Calculations were run on a PC with a 2.1 GHz-i3 processor and 4 GB RAM. The results were visualized employing PyMOL (version 1.8, Schrödinger). The docking result for the crystallised ligand was overlayed with the co-crystal structure and the calculated exhaustive root mean squared deviation (RMSD) was 0.908, indicating a good prediction ability of our docking protocol. We decided to carry out a rigid molecular docking due to close similarity in the binding site between different crystallographic structures from different inhibitors. The selected 2,4-dihydropyrano[2,3-c]pyrazole heterocyclic scaffold could be successfully docked using the validated docking protocol for GSK3β. Positions were also inspected and compared with the score algorithm, protein interaction, hydrogen bonding, and affinity interaction energies and ordered by the energy of interaction protein-ligand. Complexes were optimized using Moloc software^67^ (www.moloc.ch) with standard force field and optimization parameters. During energy minimization the position of amino acid side chains were fixed while allowing all ligand atoms to move.

### siRNA assay

The short interfering RNA (siRNA) used to knock down human GSK3β expression and the control scrambled siRNA sequence were purchased from Thermo Fisher Scientific siRNA identifier ID s6241 for GSK3β. Briefly, AREc2 cells were seeded in 6-well plates (300,000 cells/well in 2 ml complete medium without antibiotics). We knocked down GSK3β using 25 nM of the siRNA with 2 μl DharmaFECT1 transfection reagent (GE Dharmacon, T-2001-01). 48 h later, the cells were collected and GSK-3β levels were analyzed. Another set of cells were treated with scramble or siRNA for GSK3β as described for 48 h and thereafter AREc32 cells were treated with control media (basal conditions) or compound **5c** 10 μM for 24 h more. Then cells were collected to analyze luciferase activity, HO-1 and GSK3β expression.

### Neuroprotection in the SH-SY5Y neuroblastoma cell line

A pre- and co-incubation protocol was followed. Cells were pre-incubated with the corresponding compound at 1 μM in neuroblastoma cells culture medium. After 24 h, medium was removed and replaced with 1% FBS neuroblastoma culture media containing the corresponding compound (1 μM) and the toxic stimuli, the mixture of rotenone and oligomycin A (30 μM/10 μM respectively) or okadaic acid at 20 nM. Cells were co-incubated for further 24 h with the rotenone and oligomycin A solution or 18 h with the okadaic acid solution. Control cells were incubated with the same amount of DMSO without any drug. After the co-incubation period, cell viability was assessed by the MTT-reduction method.

### Nitrite production measurement in culture medium of mixed glial cells

Glial cells were pre-incubated with increasing concentrations of compounds for 24 hours. Then, treatments were removed and cells were incubated with LPS (1 μg/mL) alone or in presence of each compound at the desired concentration. Nitrite production was assessed 18 hours later by modified Griess assay. Briefly, samples (150 μl) were mixed with dapsone (75 μl) and NEDA (75 μl), and the mixture was incubated at room temperature for 5 min. Light absorption was measured at 550 nm in a microplate reader (Labtech, Offenburg, Germany). All data were normalized to basal nitrite production, considering this value as 100% of nitrite production. IC_50_ values were calculated from dose-response curves represented as percentage of nitrite production reduction induced by the different concentrations of each compound.

### ROS production measurement

Primary glial cells were cultured in bottom transparent 96-well black plates following standard protocol. Cells were pre-incubated with the corresponding compound at the selected concentrations, for 24 hours, and therafter, co-incubated with the corresponding compound and LPS (1 μM) during 18 h more. Then, treatments were removed and cells were loaded with the fluorescent probe 2′,7′-dichlorodihydrofluorescein diacetate (H_2_DCFDA) (10 μM) for 45 minutes in serum free glial culture medium. All experiments included cells treated with culture medium alone (basal). Cells were washed twice with culture medium and fluorescence intensity was recorded in a Fluostar Optima multiwall pate reader (BMG Labtech, Offenburg, Germany) at 485/520 nm as excitation and emission wavelengths respectively, each hour, during 3 hours period. Data were normalized with respect to basal conditions that were considered as 100%.

### Quantification of TNFα levels in the culture medium of mixed glial cells

Tumor necrosis factor-alpha (TNFα) concentrations were measured by specific quantitative sandwich ELISA kits (Preprotech, R&D Systems-bioNova, Madrid, Spain) following manufacturer instructions. TNFα levels in supernatant samples, obtained after treatments with culture media (Basal), LPS or LPS co-incubated with increasing concentrations of compound **5c,** were measured following manufacturer instructions. A standard curve was generated using the OD values of standard solution of TNFα. Light absorption was measured in a microplate reader (Labtech, Offenburg, Germany).

### Western Blot

After treatment, mixed glial cells were washed with cold PBS and lysed in 100 μl ice-cold lysis buffer (1% Nonident P-40, 10% glycerol, 137 mM NaCl, 20 mM Tris HCl pH 7.5, 1 μg/ml leupeptin, 1 mM phenylmethylsulfonyl fluoride, 20 mM NaF, 1 mM sodium pyrophosphate, and 1 mM Na_3_VO_4_). Then, proteins (30 μg) from cell lysates were resolved by sodium dodecyl sulfate–polyacrylamide gel electrophoresis and transferred to Immobilon-P membranes (Millipore Ibérica SA, Madrid, Spain). Membranes were incubated with anti-iNOS (BD Transduction Laboratories, USA) at 1:1000, anti-HO-1 (Chemicon, Hampshire, UK) at 1:1000, anti-GCLc (Chemicon, Hampshire, UK) at 1:1000, anti-gp91-phox (NOX2, sc-130543, Santa Cruz, Madrid, Spain) at 1:500, Anti-GSK3β (610201, BD Transduction Laboratory) at 1:2000 and anti-β-actin at 1:100,000 (Sigma, Madrid, Spain). Peroxidase-conjugated secondary antibodies (1:10000) were used to detect proteins by enhanced chemiluminescence detected by Advance Western-blotting Detection Kit (GE Healthcare, Barcelona, Spain). Scion Image program was used to quantify band intensities corresponding to immunoblot detection of protein samples. Immunoblots correspond to a representative experiment that was repeated 4 times with similar results.

### NOX activity assay

Cells were pre-incubated with increasing concentrations of the compound. After 24 hours, treatments were removed and cells were incubated with LPS (1 μg/mL) in presence of the compound at increasing concentrations for additional 4 h. Then, treatments were removed and NADPH oxidase activity was assessed by measuring O_2_^−^ production in the presence of the substrate, NADPH (100 μM) as lucigenin-enhanced chemiluminiscence (5 μM lucigenin). No enzymatic activity was detected in the absence of NADPH. Luminescence was recorded 29 times every 1.8 seconds for each well in a luminometer, and NADPH oxidase activity was expressed as relative light units (RLU)/min normalized to basal value.

### Statistical analysis

All values are expressed as mean ± S.E.M. IC_50_ and LD_50_ values were calculated by non-linear regression analysis of individual concentration-response curves using GraphPad Prism (version 5) software (San Diego, CA, USA). Analysis of the results was performed by comparison of experimental and control data by One-way or two-way ANOVA followed by Newman-Keuls *post*-*hoc* test. Differences were considered to be statistically significant when *p* ≤ 0.05. “n” represents the number of different cultures used or enzyme inhibition assays performed.

## Additional Information

**How to cite this article:** Gameiro, I. *et al*. Discovery of the first dual GSK3b inhibitor/Nrf2 inducer. A new multitarget therapeutic strategy for Alzheimer’s disease. *Sci. Rep.* **7**, 45701; doi: 10.1038/srep45701 (2017).

**Publisher's note:** Springer Nature remains neutral with regard to jurisdictional claims in published maps and institutional affiliations.

## Supplementary Material

Supporting Information

## Figures and Tables

**Table 1:** GSK3β inhibition (IC_50_ and *K*_i_ values) and Nrf2 induction capability (CD values) for the 2,4-dihydropyrano[2,3-*c*]pyrazole derivatives 5a–t and dihydro-2*H*-pyrazole[3,4-*b*]pyridine 7.

Compound	Ar	GSK3β IC_50_ (μM)	*K*i (μM)	CD (μM)^a^
Sulforaphane		—	—	0.54 ± 0.07
SB 216763	—	0.034 ± 0.01	0.0091	>30
2	—	1.5^38^	—	—
5a	Ph	>30	—	20.5 ± 3.4
5b	2-OCH_3_Ph	16.4 ± 1.8	11.5	>30
5c	3-OCH_3_Ph	7.20 ± 0.7	5.04	11.3 ± 1.3
5d	4-OCH_3_Ph	6.60 ± 1.7	4.63	25.3 ± 2.4
5e	2-CH_3_Ph	>30	—	>30
5f	3-CH_3_Ph	21.4 ± 4.6	15.0	14.1 ± 2.1
5g	4-CH_3_Ph	17.5 ± 1.9	12.3	25.4 ± 2.5
5h	4-FPh	17.9 ± 1.5	12.5	14.6 ± 4.0
5i	2-ClPh	>30	—	23.5 ± 3.2
5j	3-ClPh	20.5 ± 8.0	14.4	5.38 ± 0.5
5k	4-ClPh	6.29 ± 2.6	4.41	8.31 ± 1.5
5l	2-BrPh	>30	—	>30
5m	4-BrPh	3.77 ± 2.0	2.64	9.37 ± 1.4
5n	2-NO_2_Ph	3.89 ± 1.9	2.72	11.3 ± 5.5
5o	3-NO_2_Ph	7.20 ± 1.6	5.04	18.9 ± 2.9
5p	4-NO_2_Ph	16.3 ± 2.8	11.4	15.0 ± 4.3
5q	2-Furyl	14.8 ± 4.8	10.4	25.2 ± 2.4
5r	2-Thienyl	>30	—	17.6 ± 8.4
5s	3-Pyridyl	>30	—	8.91 ± 2.2
5t	4-Pyridyl	8.28 ± 0.7	5.80	2.48 ± 1.4
7	Ph	18.6 ± 0.4	13.1	>100

IC_50_ values were calculated from concentration-response curves of GSK3β activity as the concentration of inhibitor producing 50% reduction of enzyme activity. All compounds were incubated at four different concentrations with the enzyme and specific activity was measured after 1 h. The IC_50_ value obtained for each compound was used to calculate the apparent equilibrium dissociation constant *K*i, using the Cheng-Prusoff equation (see experimental section)^68^,^69^. AREc32 cells were treated with increasing concentrations of the corresponding compound for 24 h and thereafter, luciferase reporter activity was measured. Data are expressed as the concentration required to double the specific luciferase reporter activity (CD).

^a^Data are means ± SEM of four different experiments in duplicate.

**Table 2:** Anti-inflammatory properties of compounds 5a–t.

Compound	R	IC_50_ nitrite reduction (μM)	IC_50_ ROS reduction (μM)
Sulforaphane	—	1.4 ± 0.3	2.84 ± 0.55
5a	Ph	>60	>60
5b	2-OCH_3_Ph	43.6 ± 6.45	>60
5c	3-OCH_3_Ph	12.9 ± 0.86	18.1 ± 5.36
5d	4-OCH_3_Ph	35.4 ± 2.57	19.9 ± 4.89
5e	2-CH_3_Ph	34.3 ± 3.84	60.0 ± 0.29
5f	3-CH_3_Ph	13.4 ± 1.44	32.6 ± 6.79
5g	4-CH_3_Ph	22.3 ± 3.82	45.1 ± 5.33
5h	4-FPh	11.3 ± 1.15	37.0 ± 4.32
5i	2-ClPh	>60	>60
5j	3-ClPh	5.11 ± 0.65	24.0 ± 2.58
5k	4-ClPh	26.0 ± 2.74	>60
5l	2-BrPh	26.7 ± 1.95	38.6 ± 7.16
5m	4-BrPh	11.4 ± 2.27	33.2 ± 7.36
5n	2-NO_2_Ph	15.8 ± 5.23	19.9 ± 4.89
5o	3-NO_2_Ph	40.8 ± 2.23	30.8 ± 4.99
5p	4-NO_2_Ph	26.7 ± 4.86	>60
5q	2-Furyl	17.5 ± 2.45	22.5 ± 7.78
5r	2-Thienyl	>60	>60
5s	3-Pyridyl	7.23 ± 1.10	15.2 ± 3.73
5t	4-Pyridyl	28.8 ± 3.61	37.8 ± 1.17

IC_50_ values of nitrite production reduction and ROS production reduction elicited by compounds **5a–t** in primary glial cultures treated with LPS (1 μg/mL) during 18 h (nitrite production) and 3 h (NOX activity derived ROS production). Primary glial cultures were treated with increasing concentrations of the corresponding compound for 24 h. Then, treatments were removed and glial cells were co-incubated with the corresponding compound at increasing concentrations and LPS (1 μg/mL) for 18 h. ROS production was measured after 24 h pre-incubation with increasing concentrations of the corresponding compound followed by 18 h of co-incubation with LPS and 3 h with the fluorescent dye. Data are expressed as the concentration required to inhibit 50% the liberation of nitrites induced by LPS (IC_50_). Data are means ± SEM of four different experiments in duplicate.
