# Supplementary material for: Discovery of the first dual GSK3β inhibitor/Nrf2 inducer. A new multitarget therapeutic strategy for Alzheimer’s disease
Source: Sci Rep. 2017 Mar 31;7:45701. doi: 10.1038/srep45701 (PMC5374710; doi:10.1038/srep45701)
Supplement: Supporting Information [file srep45701-s1.doc]

*Supporting Information*

Discovery of the first dual GSK3β inhibitor / Nrf2 inducer. A new multitarget therapeutic strategy for Alzheimer’s disease

Isabel Gameiro,^1,5,‡^ Patrycja Michalska,^1,5,‡^ Giammarco Tenti,^5^ Ángel Cores,^2^ Izaskun Buendia,^1,5^ Ana I. Rojo,^4^  Nikolaos D. Georgakopoulos,^3^ Jesús M. Hernández-Guijo, ^1^ María Teresa Ramos,^2^ Geoffrey Wells,^3^ Manuela G. López,^1,5^ Antonio Cuadrado, ^4^ J. Carlos Menéndez^2^ and Rafael León^*1,5^

^1^Instituto Teófilo Hernando y Departamento de Farmacología y Terapéutica, Facultad de Medicina. Universidad Autónoma de Madrid, 28029 Madrid, Spain.

*^2^Departamento de Química Orgánica y Farmacéutica, Facultad de Farmacia, Universidad Complutense, 28040 Madrid, Spain.*

*^3^UCL School of Pharmacy, University College London, 29/39 Brunswick Square, London WC1N 1AX (UK).*

*^4^Centro de Investigación Biomédica en Red sobre Enfermedades Neurodegenerativas  (CIBERNED), Instituto de Investigación Sanitaria La Paz (IdiPaz), Instituto de  Investigaciones Biomédicas Alberto Sols UAM-CSIC y Departamento de Bioquímica, Facultad de Medicina, Universidad Autónoma de Madrid, Madrid, Spain.*

*^5^Instituto de Investigación Sanitaria, Servicio de Farmacología Clínica, Hospital Universitario de la Princesa, 28006 Madrid, Spain. E-mail:* [*rafael.leon@inv.uam.es*](mailto:rafael.leon@inv.uam.es)

^‡^ These junior authors should be considered as first authors

**TABLE OF CONTENTS**

**1. Supporting experimental data**

1.1. Chemical yields and scope of the synthesis SI3

1.2. Nrf2 induction ability of controls and compound **7** SI4

1.3. Nr2-Keap-1 protein-protein interaction inhibition study SI5

1.4. Anti-inflammatory effect of sulforaphane SI5

1.5. Time dependent NOX activity induced by LPS SI5

1.6. LD_50_ in SH-SY5Y and HepG2 cell lines SI6

**2. Experimental protocols** SI7

2.1. Synthesis SI7

2.2. Pharmacology SI22

**3. Copies of spectra** SI34

1.1. Chemical yields and scope for the synthesis

Table SI1: Yield and scope for the synthesis of pyrano[2,3-*c*]pyrazoles 5a-t

| **Entry** | **Compound** | **Ar** | **Yield (%)** |
| --- | --- | --- | --- |
| 1 | **5a** | Ph | 86 |
| 2 | **5b** | 2-OCH_3_C_6_H_4_ | 87 |
| 3 | **5c** | 3-OCH_3_C_6_H_4_ | 83 |
| 4 | **5d** | 4-OCH_3_C_6_H_4_ | 71 |
| 5 | **5e** | 2-CH_3_C_6_H_4_ | 88 |
| 6 | **5f** | 3-CH_3_C_6_H_4_ | 84 |
| 7 | **5g** | 4-CH_3_C_6_H_4_ | 81^a^ |
| 8 | **5h** | 4-FC_6_H_4_ | 73 |
| 9 | **5i** | 2-ClC_6_H_4_ | 85 |
| 10 | **5j** | 3-ClC_6_H_4_ | 77 |
| 11 | **5k** | 4-ClC_6_H_4_ | 88 |
| 12 | **5l** | 2-BrC_6_H_4_ | 82 |
| 13 | **5m** | 4-BrC_6_H_4_ | 92 |
| 14 | **5n** | 2-NO_2_C_6_H_4_ | 94 |
| 15 | **5o** | 3-NO_2_C_6_H_4_ | 91 |
| 16 | **5p** | 4-NO_2_C_6_H_4_ | 93 |
| 17 | **5q** | 2-furyl | 83 |
| 18 | **5r** | 2-thienyl | 78 |
| 19 | **5s** | 3-Pyridyl | 85 |
| 20 | **5t** | 4-Pyridyl | 84 |

^a^ Reaction time: 1h

**1.2.- Nrf2 induction ability of sulforaphane, SB216763 and compound 7.**

**Figure SI1:** Nrf2 induction activity of control compounds sulforaphane (A), SB216763 (B), compound **7** (C) and lithium (D) in the AREc32 cell line represented in bar graph normalized to basal conditions.

AREc32 cells were treated with increasing concentrations of the corresponding compound for 24 h and thereafter, luciferase reporter activity was measured. Data are means ± SEM of four different experiments in duplicate normalizing respect to luciferase basal activity in absence of any treatment. ***p < 0.001 compared to basal condition.

**1.3. Nrf2-Keap1 protein-protein interaction inhibition study.**

**Table SI2. Fluorescence polarization assay and differential scanning fluorimetry assay of the potential inhibition of the Nrf2-Keap1 protein-protein interaction.**

| **Compound** | **R** | **Nrf2 (CD_2_)**  (μM) | **FP % Inhibition**  (± SEM) | ** Δ*T_m_***  (± SEM) |
| --- | --- | --- | --- | --- |
| **Reference** | - | - | 98.67 ± 2.46 | 17.79 ± 0.61 |
| **5c** | 3-OCH_3_C_6_H_4_ | 11.3 ± 1.30 | < 0 | -0.43 ± 0.49 |
| **5m** | 4-BrC_6_H_4_ | 9.37 ± 1.41 | < 0 | -0.14 ± 0.35 |
| **5t** | 4-Pyridyl | 2.48 ± 1.43 | 1.24 ± 3.22 | -0.53 ± 0.28 |

The reference compound (“Reference”) was 2,2'-(naphthalene-1,4-diylbis(((4-methoxyphenyl)sulfonyl)azanediyl))diacetic acid described by Jiang et al.^1^ For the FP and differential scanning fluorimetry measurements a ligand concentration of 10 µM was used. Data are presented the means ± SEM of two experiments performed in triplicate.

**1.4. Anti-inflammatory effect.**

**Figure SI3:** Inhibition of nitrite production by control compound sulforaphane represented as bar graphs of the total production of nitrites normalized to basal conditions (100%).

Primary glial cell cultures were treated with increasing concentrations of the corresponding compound for 24 h, thereafter, treatments were removed and glial cells were co-incubated with each compound at increasing concentrations and LPS (1 μg/mL) for 18 h more. Data are represented as means ± SEM of four different experiments in triplicate. % of production was calculated normalizing to basal nitrite production in absence of any treatment (100 %). ^###^p < 0.001 compared to basal conditions; ***p < 0.001; **p < 0.01 compared to LPS.

**1.5.- Time-dependent NOX activity induced by LPS.**

**Figure SI4:** NOX activity induced by LPS stimulation (1 μg/mL) after 1, 2, 3 and 4 h incubation measured as superoxide production by lucigenin enhanced chemiluminiscence, in primary glial cultures represented in bar graph normalized to basal conditions.


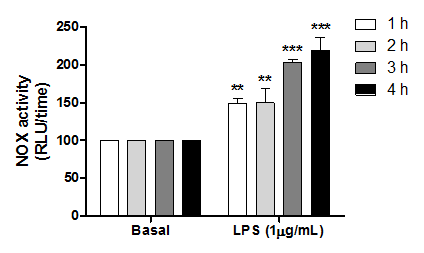


Primary glial cell cultures were treated with LPS (1 μg/mL) for 1, 2, 3 and 4 h. Thereafter, superoxide production was measured as an indirect assessment of NOX activity by the lucigenin method. Data are represented as means ± SEM of four different experiments in triplicate. % of production was calculated normalizing to basal superoxide production (100 %). ***p < 0.001; **p < 0.01 compared to basal conditions.

**1.6. LD_50_ in SH-SY5Y and HepG2 cell lines**

**SI Table 3:** Cytotoxicity elicited by compounds **5a-t** in the neuroblastoma cell line SH-SY5Y and hepatotoxicity in the hepatocarcinoma HepG2 cell line. Viability was measured as MTT reduction in presence of increasing concentrations of derivatives. Values are expressed as LD_50_ calculated from dose-response curves of four different concentrations. Data are expressed as mean ± SEM of five different experiments in triplicate.

| **Compound** | **R** | **SH-SY5Y**  **LD_50_ (µM)** | **HepG2**  **LD_50_ (μM)** |
| --- | --- | --- | --- |
| **Sulforaphane** | - | 13.27^2^ | 9.94 ± 0.7^3^ |
| **Melatonin** | - | > 100 | > 100 |
| **SB216763** | - | > 100 | > 100 |
| **5a** | Ph | > 100 | > 100 |
| **5b** | 2-OCH_3_C_6_H_4_ | > 100 | > 100 |
| **5c** | 3-OCH_3_C_6_H_4_ | > 100 | > 100 |
| **5d** | 4-OCH_3_C_6_H_4_ | > 100 | > 100 |
| **5e** | 2-CH_3_C_6_H_4_ | > 100 | > 100 |
| **5f** | 3-CH_3_C_6_H_4_ | > 100 | > 100 |
| **5g** | 4-CH_3_C_6_H_4_ | > 100 | > 100 |
| **5h** | 4-FC_6_H_4_ | > 100 | > 100 |
| **5i** | 2-ClC_6_H_4_ | > 100 | > 100 |
| **5j** | 3-ClC_6_H_4_ | > 100 | > 100 |
| **5k** | 4-ClC_6_H_4_ | > 100 | > 100 |
| **5l** | 2-BrC_6_H_4_ | > 100 | > 100 |
| **5m** | 4-BrC_6_H_4_ | > 100 | > 100 |
| **5n** | 2-NO_2_C_6_H_4_ | > 100 | > 100 |
| **5o** | 3-NO_2_C_6_H_4_ | > 100 | > 100 |
| **5p** | 4-NO_2_C_6_H_4_ | > 100 | > 100 |
| **5q** | 2-furyl | > 100 | > 100 |
| **5r** | 2-thienyl | > 100 | > 100 |
| **5s** | 3-pyridyl | > 100 | > 100 |
| **5t** | 4-pyridyl | > 100 | > 100 |

**2. EXPERIMENTAL PROTOCOLS**

**2.1. SYNTHESIS**

**Synthesis of 5-methyl-1*H*-pyrazol-3(2*H*)-one (3)**^4^

To a solution of ethyl acetoacetate (1.05 equiv, 10.5 mmol) in ethanol (15 mL) at 0 °C was added dropwise hydrazine monohydrate (1 equiv, 10 mmol). The mixture was allowed to warm to room temperature and then stirring was continued for 1 hour. After this time the solvent was removed *in vacuuo* and the solid residue was purified by crystallization. White solid, mp 223-224 °C. Spectroscopic data were in agreement with previously reported data.

### 6-Amino-3-methyl-4-phenyl-2,4-dihydropyrano[2,3-*c*]pyrazole-5-carbonitrile (5a)^5^

General procedure, prepared from 5-methyl-1*H*-pyrazol-3(2*H*)-one (98 mg, 1 mmol), malononitrile (66 mg, 1 mmol), benzaldehyde (106 mg, 1 mmol) and ammonium acetate (77 mg, 1 mmol), 5 h, crystallization from EtOH to afford compound **5a** as a white solid (0.217 g, 86% yield); Rf 0.47 (DCM:MeOH 8%); Mp: 249-250 °C (dec.); ^1^H NMR (250 MHz, DMSO-*d_6_*) δ 1.78 (s, 3H, C-3C*H*_3_), 4.59 (s, 1H, C*H*-4), 6.90 (s, 2H, C-6N*H*_2_), 7.11-7.27 (m, 3H, Ar*H*), 7.27-7.38 (m, 2H, Ar*H*), 12.11 (s, 1H, N*H*-2) ppm; ^13^C NMR (63 MHz, DMSO-*d_6_*) δ 160.91 (*C*-6), 154.80 (*C*-7a), 144.50 (C-4*C*Ar), 135.61 (*C*-3), 128.49 (2x*C*HAr), 127.52 (2x*C*HAr), 126.79 (*C*HAr), 120.88 (*C*N), 97.68 (*C*-3a), 57.15 (*C*-5), 36.25 (*C*H-4), 9.79 (C-3*C*H_3_) ppm; IR (neat) ν 3368, 3164, 2191, 1647 cm^-1^; HRMS (API-ES+) m/z: Cal. for C_14_H_12_N_4_O: 252.1011; found: [(M+H)^+^] 253.1086; [(M+Na)^+^] 275.0909; elemental analysis calcd (%) for C_14_H_12_N_4_O: C 66.65, H 4.79, N 22.21; found: C 66.43, H 4.82, N 22.08.

### 6-Amino-4-(2-methoxyphenyl)-3-methyl-2,4-dihydropyrano[2,3-*c*]pyrazole-5-carbonitrile (5b)^5^

According to the general procedure, prepared from 5-methyl-1*H*-pyrazol-3(2*H*)-one (98 mg, 1 mmol), malononitrile (66 mg, 1 mmol), 2-methoxybenzaldehyde (136 mg, 1 mmol) and ammonium acetate (77 mg, 1 mmol), 5 h, crystallization from EtOH to afford compound **5b** as a grey solid (0.245 g, 87% yield); Rf 0.40 (DCM:MeOH 8%); Mp: 235-236 °C (dec.); ^1^H NMR (250 MHz, DMSO-*d_6_*) δ 1.78 (s, 3H, C-3C*H*_3_), 3.78 (s, 3H, OC*H*_3_), 4.96 (s, 1H, C*H*-4), 6.80 (s, 2H, C-6N*H*_2_), 6.89 (t, *J*=7.3 Hz, 1H, Ar*H*), 6.94-7.04 (m, 2H, Ar*H*), 7.19 (t, *J*=7.6 Hz, 1H, Ar*H*), 12.00 (s, 1H, N*H*-2) ppm; ^13^C NMR (63 MHz, DMSO-*d_6_*) δ 161.51 (*C*-6), 156.36 (*C*-7a*), 155.09 (Ar*C*OCH_3_*), 135.08 (*C*-3), 132.13 (C-4*C*Ar), 128.65 (*C*HAr), 127.95 (*C*HAr), 120.96 (*C*N), 120.84 (*C*HAr), 111.30 (*C*HAr), 97.85 (*C*-3a), 56.33 (ArCO*C*H_3_), 55.59 (*C*-5), 29.15 (*C*H-4), 9.55 (C-3*C*H_3_) ppm; IR (neat) ν 3371, 3151, 2192, 1654, 1256 cm^-1^; HRMS (API-ES+) m/z: Cal. for C_15_H_14_N_4_O_2_: 282.1117; found: [(M+H)^+^] 283.1195; [(M+Na)^+^] 305.1039; elemental analysis calcd (%) for C_15_H_14_N_4_O_2_: C 63.82, H 5.00, N 19.85; found: C 63.52, H 5.08, N 19.59.

### 6-Amino-4-(3-methoxyphenyl)-3-methyl-2,4-dihydropyrano[2,3-*c*]pyrazole-5-carbonitrile (5c)^6^

According to the general procedure, prepared from 5-methyl-1*H*-pyrazol-3(2*H*)-one (98 mg, 1 mmol), malononitrile (66 mg, 1 mmol), 3-methoxybenzaldehyde (136 mg, 1 mmol) and ammonium acetate (77 mg, 1 mmol), 5 h, flash chromatography on silica gel dichloromethane:methanol (from 100:0 to 95:5, v/v) to afford compound **5c** as a pale yellow solid (0.235 g, 83% yield); Rf 0.40 (DCM:MeOH 8%); Mp: 201-202 °C (dec.); ^1^H NMR (250 MHz, DMSO-*d_6_*) δ 1.81 (s, 3H, C-3C*H*_3_), 3.72 (s, 3H, OC*H*_3_), 4.57 (s, 1H, C*H*-4), 6.69-6.75 (m, 2H, Ar*H*), 6.80 (dd, *J*=8.0, 1.8 Hz, 1H, Ar*H*), 6.88 (s, 2H, C-6N*H*_2_), 7.24 (t, *J*=8.0 Hz, 1H, Ar*H*), 12.10 (s, 1H, N*H*-2) ppm; ^13^C NMR (63 MHz, DMSO-*d_6_*) δ 160.93 (*C*-6), 159.24 (Ar*C*OCH_3_*), 154.76 (*C*-7a*), 146.16 (C-4*C*Ar), 135.62 (*C*-3), 129.62 (*C*HAr), 120.85 (*C*N), 119.70 (*C*HAr), 113.51 (*C*HAr), 111.67 (*C*HAr), 97.50 (*C*-3a), 56.97 (*C*-5), 54.99 (ArCO*C*H_3_), 36.18 (*C*H-4), 9.84 (C-3*C*H_3_) ppm; IR (neat) ν 3383, 3161, 2184, 1641, 1263 cm^-1^; HRMS (API-ES+) m/z: Cal. for C_15_H_14_N_4_O_2_: 282.1117; found: [(M+H)^+^] 283.1223; [(M+Na)^+^] 305.1038; elemental analysis calcd (%) for C_15_H_14_N_4_O_2_: C 63.82, H 5.00, N 19.85; found: C 63.60, H 5.07, N 19.51.

### 6-Amino-4-(4-methoxyphenyl)-3-methyl-2,4-dihydropyrano[2,3-*c*]pyrazole-5-carbonitrile (5d)^5^

According to the general procedure, prepared from 5-methyl-1*H*-pyrazol-3(2*H*)-one (98 mg, 1 mmol), malononitrile (66 mg, 1 mmol), 4-methoxybenzaldehyde (136 mg, 1 mmol) and ammonium acetate (77 mg, 1 mmol), 5 h, flash chromatography on silica gel dichloromethane:methanol (from 100:0 to 95:5, v/v) to afford compound **5d** as a light yellow solid (0.200 g, 71% yield); Rf 0.40 (DCM:MeOH 8%); Mp: 202-203 °C (dec.); ^1^H NMR (250 MHz, DMSO-*d_6_*) δ 1.78 (s, 3H, C-3C*H*_3_), 3.73 (s, 3H, OC*H*_3_), 4.53 (s, 1H, C*H*-4), 6.83 (s, 2H, C-6N*H*_2_), 6.87 (d, *J*=8.7 Hz, 2H, Ar*H*), 7.07 (d, *J*=8.7 Hz, 2H, Ar*H*), 12.07 (s, 1H, N*H*-2) ppm; ^13^C NMR (63 MHz, DMSO-*d_6_*) δ 161.04 (*C*-6), 158.31 (Ar*C*OCH_3_*), 155.10 (*C*-7a*), 136.85 (C-4*C*Ar), 135.90 (*C*-3), 128.86 (2x*C*HAr), 121.22 (*C*N), 114.11 (2x*C*HAr), 98.24 (*C*-3a), 57.90 (*C*-5), 55.35 (ArCO*C*H_3_), 35.78 (*C*H-4), 10.12 (C-3*C*H_3_) ppm; IR (neat) ν 3231, 3122, 2190, 1639, 1258 cm^-1^; HRMS (API-ES+) m/z: Cal. for C_15_H_14_N_4_O_2_: 282.1117; found: [(M+H)^+^] 283.1200; [(M+Na)^+^] 305.1024; elemental analysis calcd (%) for C_15_H_14_N_4_O_2_: C 63.82, H 5.00, N 19.85; found: C 63.73, H 4.98, N 19.62.

### 6-Amino-3-methyl-4-(2-methylphenyl)-2,4-dihydropyrano[2,3-*c*]pyrazole-5-carbonitrile (5e)^7^

According to the general procedure, prepared from 5-methyl-1*H*-pyrazol-3(2*H*)-one (98 mg, 1 mmol), malononitrile (66 mg, 1 mmol), 2-methylbenzaldehyde (120 mg, 1 mmol) and ammonium acetate (77 mg, 1 mmol), 5 h, crystallization from EtOH to afford compound **5e** as a grey solid (0.235 g, 88% yield); Rf 0.44 (DCM:MeOH 8%); Mp: 241-242 °C (dec.); ^1^H NMR (250 MHz, DMSO-*d_6_*) δ 1.68 (s, 3H, C-3C*H*_3_), 2.28 (s, 3H, ArCC*H*_3_), 4.84 (s, 1H, C*H*-4), 6.86 (s, 2H, C-6N*H*_2_), 6.96-7.01 (m, 1H, Ar*H*), 7.09-7.18 (m, 3H, Ar*H*), 12.09 (s, 1H, N*H*-2) ppm; ^13^C NMR (63 MHz, DMSO-*d_6_*) δ 160.80 (*C*-6), 155.11 (*C*-7a), 141.95 (C-4*C*Ar), 135.39 (Ar*C*CH_3_*), 135.01 (*C*-3*), 130.54 (*C*HAr), 128.95 (*C*HAr), 126.64 (*C*HAr), 126.39 (*C*HAr), 120.81 (*C*N), 97.58 (*C*-3a), 56.69 (*C*-5), 33.02 (*C*H-4), 18.99 (ArC*C*H_3_), 9.60 (C-3*C*H_3_) ppm; IR (neat) ν 3362, 3152, 2191, 1650 cm^-1^; HRMS (API-ES+) m/z: Cal. for C_15_H_14_N_4_O: 266.1168; found: [(M+H)^+^] 267.1229; [(M+Na)^+^] 289.1055; elemental analysis calcd (%) for C_15_H_14_N_4_O: C 67.65, H 5.30, N 21.04; found: C 67.51, H 5.31, N 20.99.

### 6-Amino-3-methyl-4-(3-methylphenyl)-2,4-dihydropyrano[2,3-*c*]pyrazole-5-carbonitrile (5f)^5^

According to the general procedure, prepared from 5-methyl-1*H*-pyrazol-3(2*H*)-one (98 mg, 1 mmol), malononitrile (66 mg, 1 mmol), 3-methylbenzaldehyde (120 mg, 1 mmol) and ammonium acetate (77 mg, 1 mmol), 5 h, crystallization from EtOH to afford compound **5f** as a pale yellow solid (0.223 g, 84% yield); Rf 0.44 (DCM:MeOH 8%); Mp: 205-206 °C (dec.) ^1^H NMR (250 MHz, DMSO-*d_6_*) δ 1.78 (s, 3H, C-3C*H*_3_), 2.27 (s, 3H, ArCC*H*_3_), 4.54 (s, 1H, C*H*-4), 6.87 (s, 2H, C-6N*H*_2_), 6.92-7.00 (m, 2H, Ar*H*), 7.03 (d, *J*=7.6 Hz, 1H, Ar*H*), 7.20 (t, *J*=7.8 Hz, 1H, Ar*H*), 12.09 (s, 1H, N*H*-2) ppm; ^13^C NMR (63 MHz, DMSO-*d_6_*) δ 160.88 (*C*-6), 154.76 (*C*-7a), 144.47 (C-4*C*Ar), 137.53 (Ar*C*CH_3_*), 135.58 (*C*-3*), 128.32 (*C*HAr), 127.92 (*C*HAr), 127.49 (*C*HAr), 124.72 (*C*HAr), 120.89 (*C*N), 97.70 (*C*-3a), 57.18 (*C*-5), 36.20 (*C*H-4), 21.12 (ArC*C*H_3_), 9.81 (C-3*C*H_3_) ppm; IR (neat) ν 3366, 3175, 2191, 1646 cm^-1^; HRMS (API-ES+) m/z: Cal. for C_15_H_14_N_4_O: 266.1168; found: [(M+H)^+^] 267.1276; [(M+Na)^+^] 289.1103; elemental analysis calcd (%) for C_15_H_14_N_4_O: C 67.65, H 5.30, N 21.04; found: C 67.66, H 5.41, N 20.82.

### 6-Amino-3-methyl-4-(4-methylphenyl)-2,4-dihydropyrano[2,3-*c*]pyrazole-5-carbonitrile (5g)^7^

According to the general procedure, prepared from 5-methyl-1*H*-pyrazol-3(2*H*)-one (98 mg, 1 mmol), malononitrile (66 mg, 1 mmol), 4-methylbenzaldehyde (120 mg, 1 mmol) and ammonium acetate (77 mg, 1 mmol), 1 h, crystallization from EtOH to afford compound **5g** as a white solid (0.215 g, 81% yield); Rf 0.42 (DCM:MeOH 8%); Mp: 205-206 °C (dec.); ^1^H NMR (250 MHz, DMSO-*d_6_*) δ 1.78 (s, 3H, C-3C*H*_3_), 2.27 (s, 3H, ArCC*H*_3_), 4.54 (s, 1H, C*H*-4), 6.84 (s, 2H, C-6N*H*_2_), 7.04 (d, *J*=7.8 Hz, 2H, Ar*H*), 7.12 (d, *J*=7.8 Hz, 2H, Ar*H*), 12.08 (s, 1H, N*H*-2) ppm; ^13^C NMR (63 MHz, DMSO-*d_6_*) δ 160.78 (*C*-6), 154.77 (*C*-7a), 141.52 (C-4*C*Ar), 135.73 (Ar*C*CH_3_*), 135.54 (*C*-3*), 129.02 (2x*C*HAr), 127.38 (2x*C*HAr), 120.85 (*C*N), 97.74 (*C*-3a), 57.34 (*C*-5), 35.85 (*C*H-4), 20.66 (ArC*C*H_3_), 9.78 (C-3*C*H_3_) ppm; IR (neat) ν 3372, 3184, 2191, 1645 cm^-1^; HRMS (API-ES+) m/z: Cal. for C_15_H_14_N_4_O: 266.1168; found: [(M+H)^+^] 267.1241; [(M+Na)^+^] 289.1163; elemental analysis calcd (%) for C_15_H_14_N_4_O: C 67.65, H 5.30, N 21.04; found: C 67.55, H 5.26, N 21.03.

### 6-Amino-4-(4-fluorophenyl)-3-methyl-2,4-dihydropyrano[2,3-*c*]pyrazole-5-carbonitrile (5h)^5^

According to the general procedure, prepared from 5-methyl-1*H*-pyrazol-3(2*H*)-one (98 mg, 1 mmol), malononitrile (66 mg, 1 mmol), 4-fluorobenzaldehyde (124 mg, 1 mmol) and ammonium acetate (77 mg, 1 mmol), 5 h, flash chromatography on silica gel dichloromethane:methanol (from 100:0 to 96:4, v/v) to afford compound **5h** as a pale yellow solid (0.197 g, 73% yield); Rf 0.46 (DCM:MeOH 8%); Mp: 223-224 °C (dec.); ^1^H NMR (250 MHz, DMSO-*d_6_*) δ 1.78 (s, 3H, C-3C*H*_3_), 4.63 (s, 1H, C*H*-4), 6.91 (s, 2H, C-6N*H*_2_), 7.09-7.25 (m, 4H, Ar*H*), 12.13 (s, 1H, N*H*-2) ppm; ^13^C NMR (63 MHz, DMSO-*d_6_*) δ 160.97 (d, *J*=242.4 Hz, Ar*C*F), 160.86 (*C*-6), 154.73 (*C*-7a), 140.71 (d, *J*=3.0 Hz, C-4*C*Ar), 135.66 (*C*-3), 129.38 (d, *J*=8.2 Hz, 2x*C*HAr), 120.77 (*C*N), 115.21 (d, *J*=21.4 Hz, 2x*C*HAr), 97.52 (*C*-3a), 57.04 (*C*-5), 35.44 (*C*H-4), 9.77 (C-3*C*H_3_) ppm; ^19^F NMR (235 MHz, DMSO-*d_6_*) δ -116.64 (m) ppm; IR (neat) ν 3220, 3122, 2193, 1642 cm^-1^; HRMS (API-ES+) m/z: Cal. for C_14_H_11_FN_4_O: 270.0917; [(M+H)^+^] 271.0998; [(M+Na)^+^] 293.0814; elemental analysis calcd (%) for C_14_H_11_FN_4_O: C 62.22, H 4.10, N 20.73; found: C 62.26, H 4.15, N 20.62.

### 6-Amino-4-(2-chlorophenyl)-3-methyl-2,4-dihydropyrano[2,3-*c*]pyrazole-5-carbonitrile (5i)^5^

According to the general procedure, prepared from 5-methyl-1*H*-pyrazol-3(2*H*)-one (98 mg, 1 mmol), malononitrile (66 mg, 1 mmol), 2-chlorobenzaldehyde (141 mg, 1 mmol) and ammonium acetate (77 mg, 1 mmol), 5 h, crystallization from EtOH to afford compound **5i** as a white solid (0.243 g, 85% yield); Rf 0.47 (DCM:MeOH 8%); Mp: 253-254 °C (dec.); ^1^H NMR (250 MHz, DMSO-*d_6_*) δ 1.76 (s, 3H, C-3C*H*_3_), 5.06 (s, 1H, C*H*-4), 6.96 (s, 2H, C-6N*H*_2_), 7.11-7.37 (m, 3H, Ar*H*), 7.41 (d, *J*=7.3 Hz, 1H, Ar*H*), 12.13 (s, 1H, N*H*-2) ppm. ^13^C NMR (63 MHz, DMSO-*d_6_*) δ 161.32 (*C*-6), 154.97 (*C*-7a), 140.96 (C-4*C*Ar), 135.39 (*C*-3), 131.97 (Ar*C*Cl), 130.76 (*C*HAr), 129.52 (*C*HAr), 128.64 (*C*HAr), 127.81 (*C*HAr), 120.47 (*C*N), 96.87 (*C*-3a), 55.68 (*C*-5), 33.48 (*C*H-4), 9.57 (C-3*C*H_3_) ppm; IR (neat) ν 3385, 3156, 2187, 1650, 1045 cm^-1^; HRMS (API-ES+) m/z: Cal. for C_14_H_11_ClN_4_O: 286.0621; [(M+H)^+^] 287.0679; [(M+Na)^+^] 309,0527; [(2M+H)^+^] 573,1356; found: [(2M+Na)^+^] 595,1180; elemental analysis calcd (%) for C_14_H_11_ClN_4_O: C 58.65, H 3.87, N 19.54; found: C 58.26, H 3.91, N 19.44.

### 6-Amino-4-(3-chlorophenyl)-3-methyl-2,4-dihydropyrano[2,3-*c*]pyrazole-5-carbonitrile (5j)^5^

According to the general procedure, prepared from 5-methyl-1*H*-pyrazol-3(2*H*)-one (98 mg, 1 mmol), malononitrile (66 mg, 1 mmol), 3-chlorobenzaldehyde (141 mg, 1 mmol) and ammonium acetate (77 mg, 1 mmol), 5 h, crystallization from EtOH to afford compound **5j** as a pale yellow solid (0.220 g, 77% yield); Rf 0.47 (DCM:MeOH 8%); Mp: 205-206 °C (dec.); ^1^H NMR (250 MHz, DMSO-*d_6_*) δ 1.80 (s, 3H, C-3C*H*_3_), 4.66 (s, 1H, C*H*-4), 6.99 (s, 2H, C-6N*H*_2_), 7.14 (d, *J*=7.1 Hz, 1H, Ar*H*), 7.20 (s, 1H, Ar*H*), 7.25-7.45 (m, 2H, Ar*H*), 12.18 (s, 1H, N*H*-2) ppm; ^13^C NMR (63 MHz, DMSO-*d_6_*) δ 161.06 (*C*-6), 154.73 (*C*-7a), 147.14 (C-4*C*Ar), 135.78 (*C*-3), 133.09 (Ar*C*Cl), 130.55 (*C*HAr), 127.29 (*C*HAr), 126.90 (*C*HAr), 126.37 (*C*HAr), 120.75 (*C*N), 97.07 (*C*-3a), 56.47 (*C*-5), 35.82 (*C*H-4), 9.83 (C-3*C*H_3_) ppm; IR (neat) ν 3366, 3175, 2191, 1646, 1070 cm^-1^; HRMS (API-ES+) m/z: Cal. for C_14_H_11_ClN_4_O: 286.0621; found: [(M+H)^+^] 287.0708; [(M+Na)^+^] 309,0538; elemental analysis calcd (%) for C_14_H_11_ClN_4_O: C 58.65, H 3.87, N 19.54; found: C 58.42, H 3.86, N 19.45.

### 6-Amino-4-(4-chlorophenyl)-3-methyl-2,4-dihydropyrano[2,3-*c*]pyrazole-5-carbonitrile (5k)^5^

According to the general procedure, prepared from 5-methyl-1*H*-pyrazol-3(2*H*)-one (98 mg, 1 mmol), malononitrile (66 mg, 1 mmol), 4-chlorobenzaldehyde (141 mg, 1 mmol) and ammonium acetate (77 mg, 1 mmol), 5 h, crystallization from EtOH to afford compound **5k** as a white solid (0.252 g, 88% yield); Rf 0.49 (DCM:MeOH 8%); Mp: 229-230 °C (dec.); ^1^H NMR (250 MHz, DMSO-*d_6_*) δ 1.79 (s, 3H, C-3C*H*_3_), 4.63 (s, 1H, C*H*-4), 6.94 (s, 2H, C-6N*H*_2_), 7.19 (d, *J*=8.4 Hz, 2H, Ar*H*), 7.38 (d, *J*=8.4 Hz, 2H, Ar*H*), 12.14 (s, 1H, N*H*-2) ppm; ^13^C NMR (63 MHz, DMSO-*d_6_*) δ 160.69 (*C*-6), 154.48 (*C*-7a), 143.29 (C-4*C*Ar), 135.46 (*C*-3), 131.01 (Ar*C*Cl), 129.16 (2x*C*HAr), 128.25 (2x*C*HAr), 120.46 (*C*N), 96.98 (*C*-3a), 56.50 (*C*-5), 35.33 (*C*H-4), 9.53 (C-3*C*H_3_) ppm; IR (neat) ν 3404, 3166, 2185, 1649, 1075 cm^-1^; HRMS (API-ES+) m/z: Cal. for C_14_H_11_ClN_4_O: 286.0621; found: [(M+H)^+^] 287.0666; [(M+Na)^+^] 309,0493; elemental analysis calcd (%) for C_14_H_11_ClN_4_O: C 58.65, H 3.87, N 19.54; found: C 58.55, H 3.88, N 19.55.

### 6-Amino-4-(2-bromophenyl)-3-methyl-2,4-dihydropyrano[2,3-*c*]pyrazole-5-carbonitrile (5l)^7^

According to the general procedure, prepared from 5-methyl-1*H*-pyrazol-3(2*H*)-one (98 mg, 1 mmol), malononitrile (66 mg, 1 mmol), 2-bromobenzaldehyde (185 mg, 1 mmol) and ammonium acetate (77 mg, 1 mmol), 5 h, crystallization from EtOH to afford compound **5l** as a white solid (0.271 g, 82% yield); Rf 0.49 (DCM:MeOH 8%); Mp: 248-249 °C (dec.); ^1^H NMR (250 MHz, DMSO-*d_6_*) δ 1.76 (s, 3H, C-3C*H*_3_), 5.07 (s, 1H, C*H*-4), 6.97 (s, 2H, C-6N*H*_2_), 7.07-7.27 (m, 2H, Ar*H*), 7.35 (t, *J*=7.4 Hz, 1H, Ar*H*), 7.58 (d, *J*=7.7 Hz, 1H, Ar*H*), 12.15 (s, 1H, N*H*-2) ppm; ^13^C NMR (63 MHz, DMSO-*d_6_*) δ 161.24 (*C*-6), 154.91 (*C*-7a), 142.60 (C-4*C*Ar), 135.42 (*C*-3), 132.66 (*C*HAr), 130.99 (*C*HAr), 128.95 (*C*HAr), 128.45 (*C*HAr), 122.44 (Ar*C*Br), 120.38 (*C*N), 97.06 (*C*-3a), 55.92 (*C*-5), 35.86 (*C*H-4), 9.71 (C-3*C*H_3_) ppm; IR (neat) ν 3386, 3142, 2187, 1651, 1047 cm^-1^; HRMS (API-ES+) m/z: Cal. for C_14_H_11_BrN_4_O: 330.0116; found: [(M+H)^+^] 331.0198; [(M+Na)^+^] 353,0007; elemental analysis calcd (%) for C_14_H_11_BrN_4_O: C 50.77, H 3.35, N 16.92; found: C 50.61, H 3.34, N 16.81.

### 6-Amino-4-(4-bromophenyl)-3-methyl-2,4-dihydropyrano[2,3-*c*]pyrazole-5-carbonitrile (5m)^7^

According to the general procedure, prepared from 5-methyl-1*H*-pyrazol-3(2*H*)-one (98 mg, 1 mmol), malononitrile (66 mg, 1 mmol), 4-bromobenzaldehyde (185 mg, 1 mmol) and ammonium acetate (77 mg, 1 mmol), 5 h, crystallization from EtOH to afford compound **5m** as a pale yellow solid (0.305 g, 92% yield); Rf 0.50 (DCM:MeOH 8%); Mp: 232-233 °C (dec.); ^1^H NMR (250 MHz, DMSO-*d_6_*) δ 1.79 (s, 3H, C-3C*H*_3_), 4.62 (s, 1H, C*H*-4), 6.95 (s, 2H, C-6N*H*_2_), 7.14 (d, *J*=8.4 Hz, 2H, ArC*H*), 7.51 (d, *J*=8.4 Hz, 2H, ArC*H*), 12.15 (s, 1H, N*H*-2) ppm. ^13^C NMR (63 MHz, DMSO-*d_6_*) δ 160.94 (*C*-6), 154.72 (*C*-7a), 143.95 (C-4*C*Ar), 135.70 (*C*-3), 131.41 (2x*C*HAr), 129.78 (2x*C*HAr), 120.70 (Ar*C*Br*), 119.78 (*C*N*), 97.15 (*C*-3a), 56.64 (*C*-5), 35.63 (*C*H-4), 9.78 (C-3*C*H_3_) ppm. IR (neat) ν 3392, 3177, 2188, 1638, 1073 cm^-1^; HRMS (API-ES+) m/z: Cal. for C_14_H_11_BrN_4_O: 330.0116; found: [(M+H)^+^] 331.0196; [(M+Na)^+^] 352,9992; elemental analysis calcd (%) for C_14_H_11_BrN_4_O: C 50.77, H 3.35, N 16.92; found: C 50.78, H 3.46, N 17.03.

### 6-Amino-3-methyl-4-(2-nitrophenyl)-2,4-dihydropyrano[2,3-*c*]pyrazole-5-carbonitrile (5n)^5^

According to the general procedure, prepared from 5-methyl-1*H*-pyrazol-3(2*H*)-one (98 mg, 1 mmol), malononitrile (66 mg, 1 mmol), 2-nitrobenzaldehyde (124 mg, 1 mmol) and ammonium acetate (77 mg, 1 mmol), 5 h, crystallization from EtOH to afford compound **5n** as a pale yellow solid (0.280 g, 94% yield); Rf 0.42 (DCM:MeOH 8%); Mp: 228-229 °C (dec.); ^1^H NMR (250 MHz, DMSO-*d_6_*) δ 1.76 (s, 3H, C-3C*H*_3_), 5.09 (s, 1H, C*H*-4), 7.04 (s, 2H, C-6N*H*_2_), 7.32 (dd, *J*=7.8, 1.0 Hz, 1H, Ar*H*), 7.49 (dt, *J*=8.0, 1.0 Hz, 1H, Ar*H*), 7.66 (dt, *J*=7.6, 1.0 Hz, 1H, Ar*H*), 7.85 (dd, *J*=8.1, 1.0 Hz, 1H, Ar*H*), 12.21 (s, 1H, N*H*-2) ppm; ^13^C NMR (63 MHz, DMSO-*d_6_*) δ 161.18 (*C*-6), 154.96 (*C*-7a), 149.15 (Ar*C*NO_2_), 137.62 (C-4*C*Ar*), 135.72 (*C*-3*), 133.40 (*C*HAr), 131.29 (*C*HAr), 128.35 (*C*HAr), 123.60 (*C*HAr), 120.26 (*C*N), 96.39 (*C*-3a), 55.99 (*C*-5), 31.39 (*C*H-4), 9.51 (C-3*C*H_3_) ppm; IR (neat) ν 3409, 3159, 2184, 1650, 1593, 1345 cm^-1^; HRMS (API-ES+) m/z: Cal. for C_14_H_11_N_5_O_3_: 297,0862; found: [(M+H)^+^] 298,0968; [(M+Na)^+^] 320,0784; [(2M+H)^+^] 595,1886; elemental analysis calcd (%) for C_14_H_11_N_5_O_3_: C 56.56, H 3.73, N 23.56; found: C 56.37, H 3.79, N 23.39.

### 6-Amino-3-methyl-4-(3-nitrophenyl)-2,4-dihydropyrano[2,3-*c*]pyrazole-5-carbonitrile (5o)^6^

According to the general procedure, prepared from 5-methyl-1*H*-pyrazol-3(2*H*)-one (98 mg, 1 mmol), malononitrile (66 mg, 1 mmol), 3-nitrobenzaldehyde (124 mg, 1 mmol) and ammonium acetate (77 mg, 1 mmol), 5 h, crystallization from EtOH to afford compound **5o** as a pale yellow solid (0.270 g, 91% yield); Rf 0.44 (DCM:MeOH 8%); Mp: 226-227 °C (dec.); ^1^H NMR (250 MHz, DMSO*-d_6_*) δ 1.80 (s, 3H, C-3C*H*_3_), 4.88 (s, 1H, C*H*-4), 7.06 (s, 2H, C-6N*H*_2_), 7.59-7.72 (m, 2H, Ar*H*), 8.03 (s, 1H, Ar*H*), 8.12 (dt, *J*=6.5, 2.4 Hz, 1H, Ar*H*), 12.21 (s, 1H, N*H*-2) ppm; ^13^C NMR (63 MHz, DMSO-*d_6_*) δ 161.51 (*C*-6), 155.05 (*C*-7a), 148.23 (Ar*C*NO_2_*), 147.20 (C-4*C*Ar*), 136.27 (*C*-3), 134.78 (*C*HAr), 130.64 (*C*HAr), 122.37 (*C*HAr), 122.22 (*C*HAr), 120.91 (*C*N), 97.03 (*C*-3a), 56.44 (*C*-5), 35.98 (*C*H-4), 10.13 (C-3*C*H_3_) ppm; IR (neat) ν 3412, 3162, 2193, 1644, 1593, 1345 cm^-1^; HRMS (API-ES+) m/z: Cal. for C_14_H_11_N_5_O_3_: 297.0862; found: [(M+H)^+^] 298.0896; found: [(M+Na)^+^] 320,0711; [(2M+H)^+^] 595,1766; [(2M+Na)^+^] 617,1568; elemental analysis calcd (%) for C_14_H_11_N_5_O_3_: C 56.56, H 3.73, N 23.56; found: C 56.16, H 3.80, N 23.27.

### 6-Amino-3-methyl-4-(4-nitrophenyl)-2,4-dihydropyrano[2,3-*c*]pyrazole-5-carbonitrile (5p)^5^

According to the general procedure, prepared from 5-methyl-1*H*-pyrazol-3(2*H*)-one (98 mg, 1 mmol), malononitrile (66 mg, 1 mmol), 4-nitrobenzaldehyde (124 mg, 1 mmol) and ammonium acetate (77 mg, 1 mmol), 5 h, crystallization from EtOH to afford compound **5p** as a pale yellow solid (0.275 g, 93% yield); Rf 0.42 (DCM:MeOH 8%); Mp: 279-280 °C (dec.); ^1^H NMR (250 MHz, DMSO-*d_6_*) δ 1.80 (s, 3H, C-3C*H*_3_), 4.83 (s, 1H, C*H*-4), 7.06 (s, 2H, C-6N*H*_2_), 7.46 (d, *J*=8.7 Hz, 2H, Ar*H*), 8.21 (d, *J*=8.7 Hz, 2H, Ar*H*), 12.21 (s, 1H, N*H*-2) ppm; ^13^C NMR (63 MHz, DMSO-*d_6_*) δ 161.18 (*C*-6), 154.70 (*C*-7a), 152.15 (Ar*C*NO_2_), 146.41 (C-4*C*Ar), 135.92 (*C*-3), 128.88 (2x*C*HAr), 123.95 (2x*C*HAr), 120.55 (*C*N), 96.59 (*C*-3a), 55.90 (*C*-5), 35.90 (*C*H-4), 9.78 (C-3*C*H_3_) ppm; IR (neat) ν 3410, 3219, 2194, 1640, 1511, 1347 cm^-1^; HRMS (API-ES+) m/z: Cal. for C_14_H_11_N_5_O_3_: 297.0862; found: [(M+H)^+^] 298.0951; [(M+Na)^+^] 320,0757; elemental analysis calcd (%) for C_14_H_11_N_5_O_3_: C 56.56, H 3.73, N 23.56; found: C 56.40, H 3.79, N 23.21.

### 6-Amino-4-(furan-2-yl)-3-methyl-2,4-dihydropyrano[2,3-*c*]pyrazole-5-carbonitrile (5q)^5^

According to the general procedure, prepared from 5-methyl-1*H*-pyrazol-3(2*H*)-one (98 mg, 1 mmol), malononitrile (66 mg, 1 mmol), 2-furancarboxaldehyde (96 mg, 1 mmol) and ammonium acetate (77 mg, 1 mmol), 5 h, crystallization from EtOH to afford compound **5q** as a grey solid (0.200 g, 83% yield); Rf 0.42 (DCM:MeOH 8%); Mp: > 300 °C (dec.); ^1^H NMR (250 MHz, DMSO-*d_6_*) δ 1.97 (s, 3H, C-3C*H*_3_), 4.77 (s, 1H, C*H*-4), 6.17 (d, *J*=3.0 Hz, 1H, Ar*H*), 6.37 (dd, *J*=3.0, 1.9 Hz, 1H, Ar*H*), 6.96 (s, 2H, C-6N*H*_2_), 7.53 (s, 1H, Ar*H*), 12.16 (s, 1H, N*H*-2) ppm; ^13^C NMR (63 MHz, DMSO-*d_6_*) δ 161.51 (*C*-6), 155.74 (*C*-7a*), 154.84 (C-4*C*Ar*), 142.32 (*C*HAr), 135.87 (*C*-3), 120.65 (*C*N), 110.28 (*C*HAr), 105.68 (*C*HAr), 95.14 (*C*-3a), 53.97 (*C*-5), 29.83 (*C*H-4), 9.62 (C-3*C*H_3_) ppm; IR (neat) ν 3345, 3162, 2185, 1647 cm^-1^; HRMS (API-ES+) m/z: Cal. for C_12_H_10_N_4_O_2_: 242.0804; found: [(M+H)^+^] 243.0861; [(M+Na)^+^] 265,0697; [(2M+Na)^+^] 507,1506; elemental analysis calcd (%) for C_12_H_10_N_4_O_2_: C 59.50, H 4.16, N 23.13; found: C 59.31, H 4.20, N 23.47.

### 6-Amino-3-methyl-4-(thiophen-2-yl)-2,4-dihydropyrano[2,3-*c*]pyrazole-5-carbonitrile (5r)^5^

According to the general procedure, prepared from 5-methyl-1*H*-pyrazol-3(2*H*)-one (98 mg, 1 mmol), malononitrile (66 mg, 1 mmol), 2-thiophencarboxaldehyde (112 mg, 1 mmol) and ammonium acetate (77 mg, 1 mmol), 5 h, crystallization from EtOH to afford compound **5r** as a pale yellow solid (0.201 g, 78% yield); Rf 0.43 (DCM:MeOH 8%); Mp: 243-244 °C (dec.); ^1^H NMR (250 MHz, DMSO-*d_6_*) δ 1.91 (s, 3H, C-3C*H*_3_), 4.99 (s, 1H, C*H*-4), 6.73-7.11 (m, 4H, Ar*H* and C-6N*H*_2_), 7.38 (d, *J*=4.9 Hz, 1H, Ar*H*), 12.18 (s, 1H, N*H*-2) ppm; ^13^C NMR (63 MHz, DMSO-*d_6_*) δ 161.01 (*C*-6), 154.65 (*C*-7a), 150.15 (C-4*C*Ar), 136.39 (*C*-3), 126.88 (*C*HAr), 125.37 (*C*HAr), 124.74 (*C*HAr), 121.00 (*C*N), 97.92 (*C*-3a), 57.89 (C-5), 31.75 (*C*H-4), 10.11 (C-3*C*H_3_) ppm; IR (neat) ν 3339, 3160, 2189, 1646 cm^-1^; HRMS (API-ES+) m/z: Cal. for C_12_H_10_N_4_OS: 258.0575; found: [(M+H)^+^] 259.0665; found: [(M+Na)^+^] 281,0496; [(2M+Na)^+^] 539,1085; elemental analysis calcd (%) for C_12_H_10_N_4_OS: C 55.80, H 3.90, N 21.69, S 12.41; found: C 55.45, H 3.82, N 21.46, S 12.27.

### 6-Amino-3-methyl-4-(pyridin-3-yl)-2,4-dihydropyrano[2,3-*c*]pyrazole-5-carbonitrile (5s)^6^

According to the general procedure, prepared from 5-methyl-1*H*-pyrazol-3(2*H*)-one (98 mg, 1 mmol), malononitrile (66 mg, 1 mmol), 3-pyridinecarboxaldehyde (107 mg, 1 mmol) and ammonium acetate (77 mg, 1 mmol), 5 h, crystallization from EtOH to afford compound **5s** as a grey solid (0.215 g, 85% yield); Rf 0.20 (DCM:MeOH 8%); Mp: 223-224 °C (dec.); ^1^H NMR (250 MHz, DMSO-*d_6_*) δ 1.78 (s, 3H, C-3C*H*_3_), 4.69 (s, 1H, C*H*-4), 7.01 (s, 2H, C-6N*H*_2_), 7.35 (dd, *J*=7.9, 4.8 Hz, 1H, Ar*H*), 7.52 (dd, *J*=7.9, 1.7 Hz, 1H, Ar*H*), 8.45 (s, 2H, Ar*H*), 12.19 (s, 1H, N*H*-2) ppm; ^13^C NMR (63 MHz, DMSO-*d_6_*) δ 161.11 (*C*-6), 154.78 (*C*-7a), 148.82 (*C*HAr), 148.32 (*C*HAr), 139.78 (C-4*C*Ar), 135.74 (*C*-3), 135.23 (*C*HAr), 123.95 (*C*HAr), 120.71 (*C*N), 96.82 (*C*-3a), 56.23 (*C*-5), 33.68 (*C*H-4), 9.77 (C-3*C*H_3_) ppm; IR (neat) ν 3388, 3165, 2190, 1644 cm^-1^; HRMS (API-ES+) m/z: Cal. for C_13_H_11_N_5_O: 253.0964; found: [(M+H)^+^] 254.1037; [(M+Na)^+^] 276,0859; elemental analysis calcd (%) for C_13_H_11_N_5_O: C 61.65, H 4.38, N 27.65; found: C 61.63, H 4.28, N 27.48.

### 6-Amino-3-methyl-4-(pyridin-4-yl)-2,4-dihydropyrano[2,3-*c*]pyrazole-5-carbonitrile (5t)^6^

According to the general procedure, prepared from 5-methyl-1*H*-pyrazol-3(2*H*)-one (98 mg, 1 mmol), malononitrile (66 mg, 1 mmol), 4-pyridinecarboxaldehyde (107 mg, 1 mmol) and ammonium acetate (77 mg, 1 mmol), 5 h, crystallization from EtOH to afford compound **5t** as a white solid (0.212 g, 84% yield); Rf 0.20 (DCM:MeOH 8%); Mp: 224-225 °C (dec.); ^1^H NMR (250 MHz, DMSO-*d_6_*) δ 1.81 (s, 3H, C-3C*H*_3_), 4.66 (s, 1H, C*H*-4), 7.04 (s, 2H, C-6N*H*_2_), 7.19 (d, *J*=5.8 Hz, 2H, Ar*H*), 8.51 (d, *J*=5.8 Hz, 2H, Ar*H*), 12.20 (s, 1H, N*H*-2) ppm; ^13^C NMR (63 MHz, DMSO-*d_6_*) δ 161.26 (*C*-6), 154.76 (*C*-7a), 152.86 (C-4*C*Ar), 149.95 (2x*C*HAr), 135.82 (*C*-3), 122.79 (2x*C*HAr), 120.55 (*C*N), 96.26 (*C*-3a), 55.56 (*C*-5), 35.54 (*C*H-4), 9.76 (C-3*C*H_3_) ppm; IR (neat) ν 3315, 3025, 2169, 1657 cm^-1^; HRMS (API-ES+) m/z: Cal. for C_13_H_11_N_5_O: 253.0964; found: [(M+H)^+^] 254.1068; elemental analysis calcd (%) for C_13_H_11_N_5_O: C 61.65, H 4.38, N 27.65; found: C 61.29, H 4.40, N 27.34.

### 6-amino-3-methyl-4-phenyl-4,7-dihydro-2*H*-pyrazolo[3,4-*b*]pyridine-5-carbonitrile (7).^8^

### A solution of 3-methyl-1*H*-pyrazol-5-amine 6 (291 mg, 3 mmol), malononitrile (198 mg, 3 mmol), benzaldehyde 4a (318 mg, 3 mmol) and dry ammonium acetate (231 mg, 3 mmol) in dry ethanol (5 mL) was heated under reflux for 16 hours. After this time the solvent was evaporated under reduced pressure and the crude residue was crystallized from EtOH to afford compound 7 as a white solid (330 mg, 44% yield). Rf 0.35 (DCM:MeOH 10%); Mp: > 300°C (dec.); ^1^H NMR (250 MHz, DMSO-*d_6_*) δ 1.75 (s, 3H, C-3C*H*_3_), 4.61 (s, 1H, C*H*-4), 5.44 (s, 2H, C-6N*H*_2_), 7.03-7.21 (m, 3H, C*H*-3’, C*H*-4’ and C*H*-5’), 7.21-7.39 (m, 2H, C*H*-2’ and C*H*-6’), 8.87 (s, 1H, N*H*-7), 11.69 (s, 1H, N*H*-1) ppm; ^13^C NMR (63 MHz, DMSO-*d_6_*) δ 153.38 (*C*-6), 146.87 (*C*-7a*), 146.17 (*C*-1’*), 134.87 (*C*-3), 128.25 (2x*C*HAr), 127.32 (2x*C*HAr), 126.20 (*C*HAr), 123.30 (*C*N), 100.53 (*C*-3a), 55.35 (*C*-5), 37.79 (*C*H-4), 9.51 (C-3*C*H_3_) ppm. IR (neat) ν 3401, 3324, 3225, 2163, 1628 cm^-1^; HRMS (API-ES+) m/z: Cal. for C_14_H_13_N_5_: 251.1171; found: [(M+H)+] 252.1250; elemental analysis calcd (%) for C_14_H_13_N_5_: C 66.92, H 5.21, N 27.87; found: C 66.83, H 5.21, N 27.69.

**2.2. PHARMACOLOGY**

Minimal essential medium (MEM), EMEM, DMEM with glutamax, fetal bovine serum (FBS), geneticin, and Hank's balanced salt solution were acquired from Life Technologies (Madrid, Spain). Rotenone, oligomycin A, okadaic acid, penicillin/streptomycin, sodium pyruvate, NEDA (N-(1-Naphthyl)ethylenediamine dihydrochloride), DAPSONE (4,4´-diamino-di-phenylsulfone), lipopolysaccharide (LPS), 2,2’-azobis(amidinopropane) dihydrochloride, (3-(4,5-dimethylthizaol-2-yl)-2,5-diphenyltetrazoliumbromide (MTT) and SB216763 were purchased from Sigma-Aldrich (Madrid, Spain). GSK3β Kinase Enzyme System (V1991), Luciferase Assay System (E1500) and Kinase-Glo Luminescent Kinase Assay (V6712) were purchased from Promega (Madison, WI, USA). GSK3β peptidic substrate was purchased from Millipore (Millipore Iberica, Madrid, Spain).

**GSK3β inhibition**

The ability of the compounds to inhibit GSK3β was evaluated using the method developed by Baki^9^ with some modifications. The tested compounds were dissolved in assay buffer, containing 40 mM Tris (pH 7.5), 20 mM MgCl_2_, 0.1 mg/ml BSA (bovine serum albumin) and 50 µM dithiothreitol (DTT), to achieve final reaction concentrations of 0.1, 1, 10 and 30 µM. Compound SB216763 was used as reference compound, at a final concentration of 180 nM. The GSK3β inhibition assay was performed in white 96-well plates, in duplicates. Firstly, 10 µl of enzyme (10 ng) were added to each well, followed by 10 µl of the compounds at the desired concentration. After 30 minutes, 20 µl of a mixture of ATP and GSK3β peptidic substrate were added to each well, achieving a final concentration of 1 µM of ATP and 25 of µM peptidic substrate. The mixture was incubated at 30 ºC for 60 minutes. Once the reaction was finished, the remaining ATP concentration was measured by adding 40 µl/well of Kinase-Glo reagent. The luminescence was measured in an Orion II microplate luminometer (Berthold, Germany) as relative light units (RLU). The luminescence recorded is proportional to the amount of ATP present in each well. GSK3β activity is proportional to the difference between the total ATP and the remaining ATP after the enzymatic reaction, activity was considered to be 100 % in the absence of an inhibitor..IC_50_ values were calculated by non-linear regression analysis of individual concentration-response curves using GraphPad Prism software (San Diego, CA, USA).

**GSK3β kinetic study: K_m_ calculation**

Apparent *K*_i_ for each compound were calculated from GSK3β K_m_ value for ATP, applying the Cheng-Prusoff equation^10,11^ in its simplified form: *K*_i_ = IC_50_/(1+([S]/K_m_)), in which S refers to the ATP concentration and Km is the Michaelis constant of the substrate for GSK3β. GSK3β K_m-ATP_ under our experimental conditions were calculated using a modified Baki method and a Lineweaver-Burk plot. GSK3β, ATP, and GSK3β peptidic substrate were dissolved in assay buffer, containing 40 mM Tris (pH 7.5), 20 mM MgCl_2_, 0.1 mg/ml BSA (bovine serum albumin) and 50 µM dithiothreitol (DTT). The assay was performed in white 96-well plates. To 10 µl of assay buffer, 10 µl (10 ng) of GSK3β (t = 60 min) or 10 µl of assay buffer (t = 0 min) were added, followed by 20 µl of a mixture of ATP and GSK3β peptidic substrate, achieving a final peptidic substrate concentration of 25 µM, and a final ATP concentration of 0, 0.1, 0.4, 1, 4 and 10 µM. The plate was incubated for 60 min at 30 °C. After this time, ATP concentration in absence (t = 0 min) and presence of GSK3β (t = 60 min) was determined by luminescence after the addition of 40 µl per well of Kinase-Glo reagent. The luminescence was measured in an Orion II microplate luminometer (Berthold, Germany) as relative light units (RLU). The luminescence detected is proportional to the ATP concentration in each well. The difference between the total ATP (t = 0 min, absence of enzyme) and the remaining ATP after the enzymatic reaction (t = 60 min) divided for the reaction time (60 min) was considered as the reaction rate (V) for each ATP concentration. To determine the K_m-ATP_ value, V and ATP concentration values were fitted to a Lineweaver-Burk plot (double reciprocal plot), using Origin software. *K*_i_ values were calculated using the Chen-Prusoff equation simplified: *K_i_* = IC_50_/(1+(S/*K_m_*)), being S the substrate concentration (ATP) in the experiment, and Km the Michaelis constant of the substrate for the enzyme (*K_m_* = 2,345 μM for ATP).

**Docking calculations**

The GSK3β crystallographic structure PDB ID 1PYX containing phosphoaminophosphonic acid-adenylate ester (AMP-PNP) as ligand, was used to perform a molecular docking. To test our system, we first performed the docking of ANP at the ATP-binding site using AutoDock Vina. In order to test our calculation conditions we performed the docking of the ATP analogue ATP-PNP into the ATP binding site of GSK3β. The docking result for the crystallised ligand was overlayed with the co-crystal structure and the calculated root mean squared deviation (RMSD) was 0.908, suggesting a good prediction ability of our docking protocol. We decided to carry out a rigid molecular docking (positions of protein atoms fixed) due to the close similarity in the binding site between different crystallographic structures with different inhibitors. The selected 2,4-dihydropyrano[2,3-*c*]pyrazole heterocyclic scaffold could be successfully docked using the validated docking protocol for GSK3β. Positions were also inspected and compared with the score algorithm, protein interaction, hydrogen bonding, and affinity interaction energies and ordered by the energy of interaction protein-ligand. Complexes were optimized using Moloc software^12^ ([www.moloc.ch](http://www.moloc.ch/)) with standard force field and optimization parameters. During energy minimization the position of amino acid side chains were fixed while allowing all ligand atoms to move. Docking results with compound **5m** are also in line with those reported previously by Chioua *et al*. for compound **2**.^13^

**AREc32 cells culture**

Breast cancer cells stably transfected to express luciferase after EpRE sequences MCF-7/AREc32 named AREc32, were kindly provided by Dr. C. Roland Wolf (University of Dundee, UK).^14^ AREc32 cells were maintained in DMEM with high glucose-glutamax, supplemented with 10 % FBS, 1 % penicillin-streptomycin (10,000 units), and geneticin (0.8 mg/ml G418) at 37 ºC and in a 5 % CO_2_- air atmosphere.^14^

**Luciferase activity: Nrf2 induction**

AREc32 cells are constitutively transfected with the pGL-8xARE luciferase reporter gene, inserted after EpRE sequences. Thus, Nrf2 translocation to the nucleus induces the activation of the EpRE sequences, expressing their target phase II antioxidant genes, and expressing luciferase at the same extent.^15^ AREc32 cells were plated in 96-well white plates (2X10^4^ cells/well). After 24 h, cells were incubated with increasing concentrations of each compound in duplicate for 24 h (dissolved in DMSO to give a final 0.1 % vol/vol concentration of vehicle). Each plate included non-treated cells as basal luciferase expression and tBHQ (10 μM) as positive control. TBHQ is a potent Nrf2 inducer used as control in the development of the AREc32 cells.^14^ Transfected clones are selected using G418. AREc32 cells express constitutively the plasmid pGL-8xARE that implements 8 copies of the EpRE sequences followed by a luciferase reporter gene. Therefore, the induction of Nrf2 is proportional to the activation of EpRE sequences, expressing luciferase at the same extent as EpRE sequences are activated. Luciferase production is then assessed by a bioluminescence assay. The Luciferase Assay System (Promega E1500), was used according to the Promega protocol and luminescence was quantified in an Orion II microplate luminometer (Berthold, Germany). Relative Light Units (RLU) were recorded for the contents of each well. Fold induction of luciferase activity was calculated by dividing the RLU obtained for the tests wells for each treatment by the average RLU obtained for the basal conditions. Data are expressed as CD values, expressing the concentration required to double the basal luciferase activity, used to quantify the Nrf2 induction potency. CD values are calculated from dose-response curves generated from fold induction of control conditions *vs.* inducer concentration and fitted by non-linear regression and data interpolated to value the 2-fold induction concentration, using GraphPad Prism software (San Diego, CA, USA).

**siRNA assay**

The short interfering RNA (siRNA) used to knock down human GSK3β expression and the control scrambled siRNA sequence were purchased from Thermo Fisher Scientific siRNA identifier ID s6241 for GSK3β. Briefly, AREc2 cells were seeded in 6-well plates (300,000 cells/well in 2 ml complete medium without antibiotics). We knocked down GSK3β using 25 nM of the siRNA with 2 μl DharmaFECT1 transfection reagent (GE Dharmacon, T-2001-01). 48h later, the cells were collected and GSK-3β levels were analyzed. Another set of cells were treated with scramble or siRNA for GSK3β as described for 48 h and thereafter AREc32 cells were treated with control media (basal conditions) or compound **5c** 10 μM for 24 h more. Then cells were collected to analyze luciferase activity, HO-1 and GSK3β expression.

**Fluorescence polarisation assay**

The FP assay was carried out as previously described.^16,17^ Inhibitors (10 µM) were plated onto untreated Corning® black 96 well plates containing a solution of the Keap1 Kelch domain (200 nM) and the fluorescent peptide FITC-β-DEETGEF-OH (1 nM) in Dulbecco’s Phosphate Buffered Saline (DPBS) at pH 7.4 (11% final DMSO concentration, 100 µL final volume). Following a 30 min incubation under slow agitation and away from light at room temperature, the plates were transferred to a PHERAstar microplate reader (BMG Labtech, Ortenberg, Germany) and the fluorescence polarisation was recorded. All measurements were recorded in triplicate.

**Differential scanning fluorimetry assay**

A solution of the Keap1 Kelch domain protein (5 µM) and detection dye SYPRO® orange (5X) in DPBS at pH 7.4 was added onto a qPCR plate containing the compound inhibitor (10 µM, 10% final DMSO concentration, 40 µL final volume). The plate was sealed using an optical foil seal and covered with aluminum foil to protect the dye from light. The plate was then transferred to a plate centrifuge and spun down briefly (200 G, 1 min, room temperature) to remove any bubbles and collect the solution at the bottom of the wells. Following a 30 min incubation at room temperature, the plate was placed into a 7500 Real time PCR machine and the fluorescence intensity at an excitation of 465 nm and emission of 580 nm was recorded during a temperature scan from 25 °C to 95 °C with a temperature ramping rate of 1 °C/min. All measurements were run in triplicate. Life Technologies software was used to set up the run on 7500 Software v.2.0.0 and the initial data processing was carried out on Protein thermal shift solution software. The raw data were exported to MS Excel and analysis was performed using a custom script provided by the Structural Genomics Consortium Oxford.^18^ The temperature range over which the protein unfolding occurred was selected and the maximum and minimum (at temperatures below the maximum intensity) fluorescence intensities within this range were determined. The processed data were fitted to the Boltzmann equation by non-linear regression using the Origin Pro software.

**SH-SY5Y neuroblastoma cells culture**

SH-SY5Y cells were cultured according to supplier directions in a 1:1 mixture of F12 (Ham 12) and Eagle’s MEM, supplemented with 15 non-essential amino-acids, 10 % heat‑inactivated FBS, 100 μg/mL streptomycin and 100 units/mL penicillin. Cells were maintained at 37 ºC in humidified atmosphere of 95 % air and 5 % CO_2_. For experimental procedures, cells were cultured in 48-well plates (1x10^5^ cells per well) cells/well or in 6-well plates (8x10^5^ cells per well). Treatments were carried out in 1% FBS medium unless other concentration was specified. Cells were used from 4 up to 13 passages.

**Neuroprotection in the SH-SY5Y neuroblastoma cell line**

A pre and co-incubation protocol was followed. Cells were pre-incubated with the corresponding compound at 1 μM in neuroblastoma cells culture medium. After 24 h, medium was removed and replaced with 1% FBS neuroblastoma culture media containing the corresponding compound at 1 µM and the toxic stimuli, namely a mixture of rotenone and oligomycin A (30 μM / 10 μM respectively) or okadaic acid at 20 nM. Cells were co-incubated for further 24 h with the rotenone and oligomycin A solution or 18 h with the okadaic acid solution. Control cells were incubated with the same amount of DMSO without any drug. Melatonin (1 μM) or SB216763 (1 μM) were used as positive control and reference compounds in the rotenone-oligomycin A or the okadaic acid models, respectively. After the co-incubation period, cell viability was assessed by the MTT-reduction method.

**ROS production measurement.**

Primary glial cells were cultured in bottom transparent 96-well black plates following standard protocol. Cells were pre-incubated with the corresponding compound at the selected concentrations, for 24 hours, and therafter, co-incubated with the corresponding compound and LPS (1 μM) during 18 h more. Then, treatments were removed and cells were loaded with the fluorescent probe 2',7'-dichlorodihydrofluorescein diacetate (H_2_DCFDA) (10 µM) for 45 minutes in serum free glial culture medium. All experiments included cells treated with culture medium alone (basal). Cells were washed twice with culture medium and fluorescence intensity was recorded in a Fluostar Optima multiwall pate reader (BMG Labtech, Offenburg, Germany) at 485/520 nm as excitation and emission wavelengths respectively, each hour, during 3 hours period. Data were normalized with respect to basal conditions that were considered as 100%.

**HepG2 hepatoblastoma cells culture**

HepG2 cells were cultured according to supplier indications in EMEM supplemented with 10 % heat‑inactivated FBS, 100 μg/mL streptomycin and 100 units/mL penicillin. Cells were maintained at 37 ºC in humidified atmosphere of 95 % air and 5 % CO_2_. For experimental procedures, cells were cultured in 96-well plates at density of 1x10^5^ cells/well. Treatments were performed in 1% FBS medium unless other concentration was specified. Cells were used from 4 up to 13 passages.

**Toxicology assay in SH-SY5Y neuroblastoma and in HepG2 hepatocarcinoma cells**

Cells were cultured in 48-well (SH-SY5Y, 1x10^5^ cells per well) or 96-well plates (HepG2, 1x10^5^ cells per well) for 24 h. Then, cells were treated with the compounds at 10, 30 and 100 µM in the corresponding culture media with 1% FBS After 24 h, cell viability was assessed by the MTT-reduction assay. Control cells were incubated with the same concentration of DMSO (0.1 % final concentration) without any drug.

**Viability assay by MTT-reduction in SH-SY5Y neuroblastoma and HepG2 hepatocarcinoma cells**

SH-SY5Y and HepG2 cells viability was measured by the MTT assay.^19^ This method is based on the cleavage of the tetrazolium ring of MTT by active dehydrogenases, generating a formazan precipitate. At the end of the neuroprotection or toxicity experiments, a MTT solution was added to each well to reach a final concentration of 0.5 mg/mL. After 2 additional hours of incubation, the solution was replaced by 300 μL of DMSO to solubilize the formazan precipitate. Absorbance was measured in a FLUOStar Optima plate reader at 540 nm. The absorbance obtained in control cells was considered as 100 % of cell survival and used to reference all treatments.

The percentage of protection was calculated as follows: the percentage of cell survival obtained with the toxic stimuli-treated cells subtracted to 100 was normalized to 100 % of cell death. The percentage of protection was obtained by subtracting the percentage of cell death in presence of the tested compound to 100%.

**Ethical issues on the use of animals**

All experimental procedures were performed following the *Guide for the Care and Use of Laboratory Animals* and were previously approved by the institutional Ethics Committee of the Autonomous University of Madrid, Spain, according to the European Guidelines for the use and care of animals for research in accordance with the European Union Directive of 22 September 2010 (2010/63/UE) and with Spanish Royal Decree of 1 February 2013 (53/2013). All efforts were made to minimize animal suffering and to reduce the number of animals used.

**Mixed glial culture**

Mixed glial cultures were obtained from cerebral cortex of 2-5 day-old Sprague-Dawley rats. After removal of blood vessels and meninges, the forebrains were dissociated in DMEM/F12 medium. After mechanical dissociation, cells were plated (3x10^5^ cells/ml) in DMEM/F12 medium with 20% fetal bovine serum (FBS) and 1 % penicillin/streptomycin (10,000 units), at 37 ºC and in a 5 % CO_2_-supplemented air atmosphere. After 5 days, medium was substituted by DMEM/F12 medium with 10 % FBS. Cells were cultured for 7-10 days before treatment.

**Nitrite production measurement in culture medium of mixed glial cells**

Primary rat glial cells were treated with compounds **5a-t** at increasing concentrations (3, 10, 30 and 60 μM) during 24 h. Thereafter, treatments were removed and glial cells were co‑incubated with increasing concentrations of each compound and LPS (1 μg/mL) for 18 h more. At the end of this period, supernatant was collected and the presence of nitrites was analyzed by the Griess method with modifications.^20^ Each plate included non-treated cells as basal nitrite production, sulforaphane (SFN, 10 μM) as positive control. Briefly, samples (150 µl) were mixed with DAPSONE (75 µl) and NEDA (75 µl), and the mixture was incubated at room temperature for 5 min. Light absorption was measured at 550 nm in a microplate reader (Labtech, Offenburg, Germany). All data were normalized to basal nitrite production, considering this value as 100% of nitrite production. EC_50_ values were calculated from dose-response curves represented as percentage of nitrite production reduction induced by the different concentrations of each compound. Concentration response curves were fitted by non-linear regression analysis of individual concentration-response curves using GraphPad Prism software (San Diego, CA, USA). EC_50_ values are expressed as mean ± SEM of four different experiments in triplicate.

**NOX activity assay**

Cells were pre-incubated with increasing concentrations of the compound. After 24 hours, treatments were removed and cells were incubated with LPS (1 μg/mL) in presence of the compound at increasing concentrations for additional 4 h. Then, treatments were removed and NADPH oxidase activity was assessed by measuring O_2_^·-^ production in the presence of the substrate, NADPH (100 μM) as lucigenin-enhanced chemiluminiscence (5 μM lucigenin). No enzymatic activity was detected in the absence of NADPH. Luminescence was recorded 29 times every 1.8 seconds for each well in a luminometer, and NADPH oxidase activity was expressed as relative light units (RLU)/min normalized to basal value.

**Quantification of TNFα levels in the culture medium of mixed glial cells**

Tumor necrosis factor-alpha (TNFα) concentrations were measured by specific quantitative sandwich ELISA kit (Preprotech, R&D Systems-bioNova, Madrid, Spain) following manufacturer instructions. Supernatant samples obtained after treatments with culture media (Basal), LPS or LPS co-incubated with increasing concentrations of compound **5c** were measured following manufacturer instructions. A standard curve was generated using the OD values of standard solution of TNFα. Light absorption was measured in a microplate reader (Labtech, Offenburg, Germany).

**Western Blot**

After treatment, mixed glial cells were washed with cold PBS and lysed in 100 µl ice-cold lysis buffer (1% Nonident P-40, 10 % glycerol, 137 mM NaCl, 20 mM Tris HCl pH 7.5, 1 μg/ml leupeptin, 1 mM phenylmethylsulfonyl fluoride, 20 mM NaF, 1 mM sodium pyrophosphate, and 1 mM Na_3_VO_4_). Then, proteins (30 µg) from cell lysates were resolved by sodium dodecyl sulfate–polyacrylamide gel electrophoresis and transferred to Immobilon-P membranes (Millipore Iberica SA, Madrid, Spain). Membranes were incubated with anti-iNOS (BD Transduction Laboratories, USA) at 1:1000, anti-HO-1 (Chemicon, Hampshire, UK) at 1:1000, anti-GCLc (Chemicon, Hampshire, UK) at 1:1000 anti-gp91-phox (NOX2, sc-130543, Santa Cruz, Madrid, Spain) at 1:500, Anti-GSK3β (610201, BD Transduction Laboratory) at 1:2000 and anti-β-actin at 1:100,000 (Sigma, Madrid, Spain). Peroxidase-conjugated secondary antibodies (1:10000) were used to detect proteins by enhanced chemiluminescence detected by Advance Western-blotting Detection Kit (GE Healthcare, Barcelona, Spain). Scion Image program was used to quantify band intensities corresponding to immunoblot detection of protein samples. Immunoblots correspond to a representative experiment that was repeated 4 times with similar results.

**Statistical analysis**

All values are expressed as mean ± S.E.M. IC_50_ and LD_50_ values were calculated by non-linear regression analysis of individual concentration-response curves using GraphPad Prism software (San Diego, CA, USA). Analysis of the results was performed by comparison between experimental and control data by One-way ANOVA followed by Newman-Keuls *post-hoc* test. Differences were considered to be statistically significant when *p* ≤ 0.05. “n” represents the number of different cultures used or enzyme inhibition assays performed.

# 3. COPIES OF SPECTRA

## 6-Amino-3-methyl-4-phenyl-2,4-dihydropyrano[2,3-*c*]pyrazole-5-carbonitrile (5a)

## 6-Amino-4-(2-methoxyphenyl)-3-methyl-2,4-dihydropyrano[2,3-*c*]pyrazole-5-carbonitrile (5b)

## 6-Amino-4-(3-methoxyphenyl)-3-methyl-2,4-dihydropyrano[2,3-*c*]pyrazole-5-carbonitrile (5c)

## 6-Amino-4-(4-methoxyphenyl)-3-methyl-2,4-dihydropyrano[2,3-*c*]pyrazole-5-carbonitrile (5d)

## 6-Amino-3-methyl-4-(2-methylphenyl)-2,4-dihydropyrano[2,3-*c*]pyrazole-5-carbonitrile (5e)

## 6-Amino-3-methyl-4-(3-methylphenyl)-2,4-dihydropyrano[2,3-*c*]pyrazole-5-carbonitrile (5f)

## 6-Amino-3-methyl-4-(4-methylphenyl)-2,4-dihydropyrano[2,3-*c*]pyrazole-5-carbonitrile (5g)

## 6-Amino-4-(4-fluorophenyl)-3-methyl-2,4-dihydropyrano[2,3-*c*]pyrazole-5-carbonitrile (5h)

## 6-Amino-4-(2-chlorophenyl)-3-methyl-2,4-dihydropyrano[2,3-*c*]pyrazole-5-carbonitrile (5i)

## 6-Amino-4-(3-chlorophenyl)-3-methyl-2,4-dihydropyrano[2,3-*c*]pyrazole-5-carbonitrile (5j)

## 6-amino-4-(4-chlorophenyl)-3-methyl-2,4-dihydropyrano[2,3-*c*]pyrazole-5-carbonitrile (5k)

## 6-Amino-4-(2-bromophenyl)-3-methyl-2,4-dihydropyrano[2,3-*c*]pyrazole-5-carbonitrile (5l)

## 6-Amino-4-(4-bromophenyl)-3-methyl-2,4-dihydropyrano[2,3-*c*]pyrazole-5-carbonitrile (5m)

## 6-Amino-3-methyl-4-(2-nitrophenyl)-2,4-dihydropyrano[2,3-*c*]pyrazole-5-carbonitrile (5n)

## 6-Amino-3-methyl-4-(3-nitrophenyl)-2,4-dihydropyrano[2,3-*c*]pyrazole-5-carbonitrile (5o)

## 6-Amino-3-methyl-4-(4-nitrophenyl)-2,4-dihydropyrano[2,3-*c*]pyrazole-5-carbonitrile (5p)

## 6-amino-4-(furan-2-yl)-3-methyl-2,4-dihydropyrano[2,3-*c*]pyrazole-5-carbonitrile (5q)

## 6-amino-3-methyl-4-(thiophen-2-yl)-2,4-dihydropyrano[2,3-*c*]pyrazole-5-carbonitrile (5r)

## 6-Amino-3-methyl-4-(pyridin-3-yl)-2,4-dihydropyrano[2,3-*c*]pyrazole-5-carbonitrile (5s)

## 6-amino-3-methyl-4-(pyridin-4-yl)-2,4-dihydropyrano[2,3-*c*]pyrazole-5-carbonitrile (5t)

## 6-amino-4-(4-chlorophenyl)-3-methyl-4,7-dihydro-1H-pyrazolo[3,4-*b*]pyridine-5-carbonitrile (7)

**SI References**

1 Jiang, Z. Y. *et al.* Discovery of potent Keap1-Nrf2 protein-protein interaction inhibitor based on molecular binding determinants analysis. *Journal of medicinal chemistry* **57**, 2736-2745, doi:10.1021/jm5000529 (2014).

2 Hu, K. *et al.* Synthesis and biological evaluation of sulforaphane derivatives as potential antitumor agents. *European journal of medicinal chemistry* **64**, 529-539, doi:10.1016/j.ejmech.2013.03.045 (2013).

3 Park, S. Y., Kim, G. Y., Bae, S. J., Yoo, Y. H. & Choi, Y. H. Induction of apoptosis by isothiocyanate sulforaphane in human cervical carcinoma HeLa and hepatocarcinoma HepG2 cells through activation of caspase-3. *Oncology reports* **18**, 181-187 (2007).

4 Jung, J., Watkins, E. & Avery, M. Synthesis of 3-substituted and 3,4-disubstituted pyrazolin-5-ones. *Tetrahedron* **58**, 3639-3646 (2002).

5 Guo, Y. *et al.* Meglumine promoted one-pot, four-component synthesis of pyranopyrazole derivatives. *Tetrahedron* **69** (2013).

6 Vasuki, V. & Kumaravel, M. Rapid four-component reactions in water: synthesis of pyranopyrazoles. *Tetrahedron Letters* **49**, 5636-5638 (2008).

7 Muramulla, S. & Zhao, C.-G. A new catalytic mode of the modularly designed organocatalysts (MDOs): enantioselective synthesis of dihydropyrano[2,3-c]pyrazoles. *Tetrahedron Letters* **52** (2011).

8 Nadia R. Mohamed, N. Y. K., Amin F. Fahmy, Ahmed A. El-Sayed. Facile synthesis of fused nitrogen containing heterocycles as anticancer agents. *Der Pharma Chemica* **2** (2010).

9 Baki, A., Bielik, A., Molnar, L., Szendrei, G. & Keseru, G. M. A high throughput luminescent assay for glycogen synthase kinase-3beta inhibitors. *Assay and drug development technologies* **5**, 75-83, doi:10.1089/adt.2006.029 (2007).

10 Cheng, Y. & Prusoff, W. H. Relationship between the inhibition constant (K1) and the concentration of inhibitor which causes 50 per cent inhibition (I50) of an enzymatic reaction. *Biochemical pharmacology* **22**, 3099-3108 (1973).

11 Kozikowski, A. P. *et al.* Structure-based design leads to the identification of lithium mimetics that block mania-like effects in rodents. possible new GSK-3beta therapies for bipolar disorders. *Journal of the American Chemical Society* **129**, 8328-8332, doi:10.1021/ja068969w (2007).

12 Gerber, P. R. & Muller, K. MAB, a generally applicable molecular force field for structure modelling in medicinal chemistry. *Journal of computer-aided molecular design* **9**, 251-268 (1995).

13 Chioua, M. *et al.* Synthesis and biological evaluation of 3,6-diamino-1H-pyrazolo[3,4-b]pyridine derivatives as protein kinase inhibitors. *Bioorganic & medicinal chemistry letters* **19**, 4566-4569, doi:10.1016/j.bmcl.2009.06.099 (2009).

14 Wang, X. J., Hayes, J. D. & Wolf, C. R. Generation of a stable antioxidant response element-driven reporter gene cell line and its use to show redox-dependent activation of nrf2 by cancer chemotherapeutic agents. *Cancer research* **66**, 10983-10994, doi:10.1158/0008-5472.CAN-06-2298 (2006).

15 Wang, X. J., Hayes, J. D. & Wolf, C. R. Generation of a stable antioxidant response element-driven reporter gene cell line and its use to show redox-dependent activation of nrf2 by cancer chemotherapeutic agents. *Cancer Res.* **66**, 10983-10994, doi:10.1158/0008-5472.CAN-06-2298 (2006).

16 Hancock, R. *et al.* Peptide inhibitors of the Keap1-Nrf2 protein-protein interaction. *Free radical biology & medicine* **52**, 444-451, doi:10.1016/j.freeradbiomed.2011.10.486 (2012).

17 Hancock, R., Schaap, M., Pfister, H. & Wells, G. Peptide inhibitors of the Keap1-Nrf2 protein-protein interaction with improved binding and cellular activity. *Organic & biomolecular chemistry* **11**, 3553-3557, doi:10.1039/c3ob40249e (2013).

18 Niesen, F. H., Berglund, H. & Vedadi, M. The use of differential scanning fluorimetry to detect ligand interactions that promote protein stability. *Nature protocols* **2**, 2212-2221, doi:10.1038/nprot.2007.321 (2007).

19 Denizot, F. & Lang, R. Rapid colorimetric assay for cell growth and survival. Modifications to the tetrazolium dye procedure giving improved sensitivity and reliability. *Journal of immunological methods* **89**, 271-277 (1986).

20 Schulz, K., Kerber, S. & Kelm, M. Reevaluation of the Griess method for determining NO/NO2- in aqueous and protein-containing samples. *Nitric oxide : biology and chemistry / official journal of the Nitric Oxide Society* **3**, 225-234, doi:10.1006/niox.1999.0226 (1999).
